# Effectiveness of a neuropsychological treatment for confabulations after brain injury: A clinical trial with theoretical implications

**DOI:** 10.1371/journal.pone.0173166

**Published:** 2017-03-03

**Authors:** Mónica Triviño, Estrella Ródenas, Juan Lupiáñez, Marisa Arnedo

**Affiliations:** 1 Department of Neuropsychology. San Rafael University Hospital, Granada, Spain; 2 Mind, Brain, and Behavior Research Center. University of Granada, Granada, Spain; 3 Department of Experimental Psychology. University of Granada, Granada, Spain; 4 Department of Psychobiology. University of Granada, Granada, Spain; Universidade Federal do Rio de Janeiro, BRAZIL

## Abstract

Confabulators consistently generate false memories without intention to deceive and with great feelings of rightness. However, to our knowledge, there is currently no known effective treatment for them. In order to fill this gap, our aim was to design a neuropsychological treatment based on current theoretical models and test it experimentally in 20 confabulators sequentially allocated to two groups: an experimental and a control group. The experimental group received nine sessions of treatment for three weeks (three sessions per week). The sessions consisted of some brief material that participants had to learn and recall at both immediate and delayed time points. After this, patients were given feedback about their performance (errors and correct responses). Pre- and post-treatment measurements were recorded. Confabulators in the control group were included in a *waiting list* for three weeks, performed the pre- and post- measurements without treatment, and only then received the treatment, after which a post-treatment measurement was recorded. This applied to only half of the participants; the other half quit the study prematurely. Results showed a significant decrease in confabulations and a significant increase in correct responses in the experimental group; by contrast, patients in the control group did not improve during the *waiting list* period. Only control group patients who subsequently received the treatment after serving as controls improved. The effects of the treatment were generalized to patients’ everyday lives, as reported by relatives, and persisted over time. This treatment seems to be effective and easy to implement and consequently of clinical interest. Moreover, it also has theoretical implications regarding the processes related to the genesis and/or maintenance of confabulations. In particular, results point to a deficit in early stages of memory retrieval with the preservation of later strategic monitoring processes. Specifically, some of the processes involved may include selective attention or early conflict detection deficits. Future research should test these hypotheses.

## Introduction

A confabulation is the generation of false memories without intention to deceive. Confabulators have a great feeling of rightness and show great resistance to being confronted with the truth, which leads them to behave according to their own confabulations [1–2]. Four types of confabulations have been proposed [3]: 1) intrusions in memory tests or simple provoked confabulations; 2) momentary confabulations or false verbal statements in a discussion or other situations; 3) fantastic confabulations, which have no basis in reality or logic; and 4) behaviorally spontaneous confabulations, according to which patients act. These mnestic confabulations are always related to personal past memories or future plans. However, there are also non-mnestic confabulations often known as paramnesic misidentification, such as reduplicative paramnesia (the mental duplication of places or people), Capgras syndrome (misidentification of people), Fregoli syndrome (hyperfamiliarity of unknown faces), and pseudohallucinations (misidentification of objects). Mnestic and non-mnestic confabulations frequently co-occur. In fact, non-mnestic confabulations have been described frequently in patients who also had behavioral or bizarre confabulations. However, they have been dissociated, suggesting differences in their neural basis and underlying cognitive mechanisms [3].

From a clinical viewpoint, confabulations have a major impact on daily functioning, as confabulators require continuous supervision and are prevented from having normal social and family lives. In addition, although confabulations are often described as a transient symptom, multiple studies have shown that some patients chronically confabulate more than one year after an injury [4–9]. As a result, it is very important to develop treatments to prevent confabulations from becoming chronic and reduce their impact on patients’ functionality as well as patients’ dependence on relatives.

However, we are only aware of two single case studies describing interventions with confabulators [10–11]. Dayus and Van den Broek [10] trained a patient to reduce the number of swear words that he said and control his many confabulations. They considered both behaviors to reflect a deficit in self-monitoring. According to their predictions, an increase in self-control over swear words (by reinforcing the patient every time he pressed a hand-held clicker when he detected a swear word) would simultaneously lead to a decrease in the number of confabulations. Their prediction was fulfilled after 51 sessions performed over three months. In another study, Del Grosso et al. [11] focused on contrasting the confabulations recorded in a diary by a patient with reality and confronting the patient with such reality. The authors considered the central deficit to be anosognosia. An improvement was recorded after 76 sessions over three months, in combination with conventional cognitive rehabilitation. The common point of both interventions appears to be the systematic feedback provided about swear words or confabulations, leaving no doubt about them; this seems to be critical, as providing non-systematic feedback seems to reinforce and favor the subsequent maintenance of confabulations.

However, although these interventions seem to be effective, they can hardly be accomplished in a standardized way for the following reasons: 1) the high frequency and long duration of the treatment, which might increase their efficiency but prevents their use in brief hospitalizations during acute and subacute phases following brain injury; 2) the difficulty of generalizing individualized procedures (e.g., the reduction of swear words) to most patients because this type of behavior is infrequent; 3) the problems with using diaries due to the comorbidity of severe memory deficits, writing disorders, illiteracy, visual problems, or anosognosia, which lead patients to consider a diary useless; 4) the difficulty in determining the effects of treatment when other therapies are administered simultaneously; and 5) the problems with generalizing the results due to the application of the treatment to single cases.

Hence, the main aim of the present study was to design a treatment inspired by the two previous interventions and focused on systematically contrasting momentary confabulations with reality, while avoiding the shortcomings mentioned above. Our treatment was reasonably short, was based on the confabulations themselves, focused on easy learning material without diaries, and did not require another simultaneous therapy. Importantly, we based the treatment on current theoretical models and tested it experimentally for the first time in a group of confabulators.

One of the most influential models of confabulations, proposed by the Schnider group, is the *reality-filtering hypothesis* [8,12–18]. This hypothesis suggests that patients fail to suppress memory traces that were previously activated but are currently irrelevant. According to the *dual monitoring deficit* proposed by the Toronto group [1–2,7,19], confabulators fail in one of two ways: either at a very early, preconscious stage of memory retrieval, showing a deficit in the mechanism that monitors the relevance of memory associations and the feeling of rightness that they generate, or in later monitoring processes, such as strategic retrieval or verification. Other models propose variations in monitoring deficits, such as a deficit in *source monitoring*, that is, the ability to locate the temporal and contextual sources of each recollection [20–21]. Recently, another approach has suggested that the core deficit is an *excessive processing of task-irrelevant information* that inflates the ‘feeling of rightness’, leading to an unsuccessful verification by later monitoring processes [6].

Based on these models, we designed a treatment and applied it to a group of patients (i.e., the experimental group). Results were compared to those of a control group of confabulators who only received the treatment after serving as controls. This intervention was expected to improve at least one or more of the following processes: 1) selective attention during the learning phase, training patients to focus on the relevant details of the stimuli that allowed them to filter the irrelevant information; 2) monitoring processes during the retrieval phase, reinforcing the strategic search processes and training patients to inhibit traces that were irrelevant for the task; and 3) memory control processes after the retrieval phase, making patients aware of their confabulations and teaching them to verify their memories before making decisions. If one or more of these processes are involved in confabulations, as proposed by the above-mentioned models, we should expect improvements among the patients. Specifically, we expected to find a significant decrease in confabulations and an improvement in all the variables related to them. Determining the mechanisms involved in confabulations was beyond the aim of the present study. Yet, we expected to observe changes in behaviors and neuropsychological variables associated with either early, preconscious mechanisms (i.e., irrelevant information processing, reality filtering impairment, or feeling of rightness deficit) or later, executive mechanisms (i.e., monitoring processes), which could be useful for understanding the role of the different processes proposed.

## Method

### Design

After obtaining the results and verifying their clinical relevance, the study was registered as a clinical trial on the ClinicalTrials.gov platform (ID: NCT02540772) in order to communicate the results to the scientific community. The authors confirm that all the ongoing and related trials required for this intervention are registered.

This trial was aimed at exploring the effectiveness of a neuropsychological treatment for confabulations for three weeks. Due to the short duration of the intervention and in order to control for spontaneous recovery—given that the time since the injury was less than six months in several patients (see below)—the treatment was compared against a control condition without treatment (i.e., *waiting list*). Participants were assigned sequentially by a neuropsychologist (the head of the San Rafael University Hospital Neuropsychology Department) to either the experimental or the control group when they were referred to our unit. For ethical reasons, treatment was also administered to participants in the control group after recording their measures for the second time, following a three-week interval without treatment. Given that some of the first patients allocated to the control group did not stay in the hospital enough time to complete the treatment, patients who enrolled later were assigned to the groups according to geographical provenance. Specifically, patients from the city of Granada were assigned to the control group (and were later given the treatment), and patients from other towns and villages were assigned to the experimental group. This allowed us to ensure that all participants received the treatment. In any case, no differences were observed between firstly and lastly allocated patients in the analyses performed subsequently.

Patients and their relatives were offered the opportunity to participate in a study in which an experimental treatment for memory impairment was being tested. They were informed that the treatment was harmless and consisted of learning and remembering different items, without specifying that our main objective was to reduce the confabulations. Moreover, no information about the possible outcomes was provided because they were currently being studied. Depending on the assigned group, both participants and their relatives were informed about one of the two procedures (i.e., treatment or *waiting list* before treatment) without mentioning the existence of the other group. Only the therapist knew the group to which each patient was assigned. The measurement and analysis of outcomes were also blinded to all the participants and their relatives, although each participant received a clinical report with his/her neuropsychological results before and after treatment (without knowing which group he/she belonged to).

**Fig 1:**
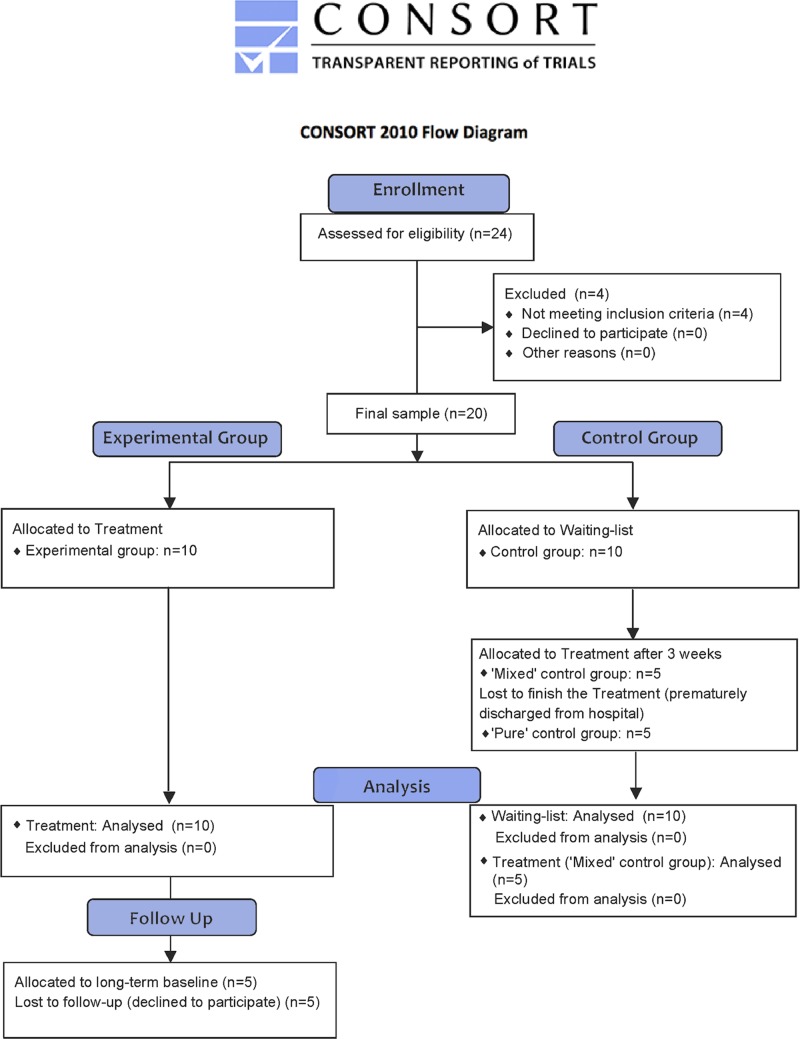
CONSORT flow diagram. The diagram shows the number of participants enrolled and those who were excluded or assigned to each of the two groups (experimental vs. control).

Repeated measures were included for both the experimental and control groups. Specifically, two measures were recorded in the experimental group (pre- and post-treatment), while three were recorded in the control group (pre- and post-waiting, and post-treatment). Five of the control patients were discharged prematurely, so the control group was divided into two subgroups: a 'pure' control group that never completed the treatment (we were not able to record the post-treatment measure) and a 'mixed' control group that completed it normally. Moreover, five patients in the experimental group completed a long-term follow-up measure (at three, nine, or 18 months after the treatment was finished). Fig 1 shows the CONSORT flowchart with the selection, allocation, and follow-up of participants. More information about the trial can be found in the CONSORT checklist included in the S1 File.

### Participants

Sample size was difficult to establish, as previous studies with the same aim used single cases. We determined that the necessary number of participants per group ranged form 6 to 10 on the basis of our previous experience with frontal patients [22–24]. Our experience showed that differences from a healthy or impaired control group could be significantly established (e.g., in reaction times, accuracy or neuropsychological tests) with an N of about 10 participants. We first collected data from six participants in each group and analyzed the data using GPower 3.1 [25] to compare the effect of treatment (pre- minus post-treatment) in the experimental group with the effect of *waiting list* (pre- minus post-waiting) in the control group for a repeated measures ANOVA. With an α value of .05 and estimated effect size of 12.10, the estimated required sample size was four participants. Consequently, we completed the sample for up to 10 participants per group to ensure that the results were not spurious.

Thus, the sample was recruited during the period between April 2013 and April 2015 at San Rafael Hospital. During this period, approximately 500 patients were treated at the Neuropsychology Service of the hospital, where they were admitted for rehabilitation after an acquired brain injury. Relatives or doctors reported the presence of spontaneous confabulations for at least three months (from injury to the first clinical interview) and without clinical improvement (interfering with the patient’s daily life with frequent arguments and exhaustive supervision). Given that the treatment was intended to provoke confabulations in order to provide feedback, the main inclusion criteria were showing momentary confabulations in the Spanish adaptation of the Dalla Barba Provoked Confabulation Interview [26–27]. This structured interview contains 60 questions organized in the following categories: episodic knowledge (e.g., what did you eat for dinner last night?), personal semantic knowledge (e.g., age, current address), general semantic knowledge (e.g., what happened to the Twin Towers?), semantic knowledge of words (e.g., what does 'bed' mean?), 'I don't know' episodic (e.g., what did you do on March 13, 1985?), 'I don't know' semantic (e.g., who won the World Cup in 1977?) and 'I don't know' non-words (e.g., what does 'adikapo' mean?). Exclusion criteria included the presence of impairment in alertness, dementia, acute confusional state, or a history of alcohol or drug abuse, as well as a history of psychiatric illness. If the main criteria were met, participants underwent a neuropsychological assessment (see below). From the original sample of 24 participants who showed spontaneous confabulations, two participants were excluded due to the absence of momentary confabulations in the Dalla Barba interview. Another two participants were excluded because of a deficit in alertness in the neuropsychological assessment (see Fig 1). Prior to their injury, all 20 patients included in the study were completely independent in their daily living and the younger patients were occupationally active.

In addition to momentary confabulations, other types of confabulations were recorded. For example, intrusions in memory were recorded in memory tests during the neuropsychological assessment (see below). Additionally, both therapists and relatives recorded the presence of behaviorally spontaneous or fantastic confabulations, as well as other non-mnestic confabulations (i.e., Fregoli syndrome, Capgras syndrome, reduplicative paramnesia, and pseudohallucinations). All the patients scored 10 or higher (18 on average) on the Dalla Barba interview and had behaviorally spontaneous confabulations. Specifically, patients without motor dysfunction tried to go out or go to work and needed supervision to prevent their escape. Those with motor deficits, such as hemiplegia, tried to walk and required mechanical restraints to prevent their falling, but their intentions were beyond the anosognosia of hemiplegia since all tried to do activities like going to work or doing housework. In fact, eight patients suffered from neglect syndrome. Fantastic confabulations (i.e., false memories apparently unrelated to past events, such as believing that the hospital is an art exhibition or that they have made incredible journeys or met famous people) were present in 12 patients, although they were sporadic in six patients. Regarding non-mnestic confabulations, all patients experienced Fregoli syndrome, 15 patients also showed reduplicative paramnesia (thus insisting on the existence of two houses, cars, rooms, hospitals, bathrooms, gyms, roommates, etc.) and 17 had pseudohallucinations. None suffered from Capgras syndrome. Information on behavioral and fantastic confabulations, related symptoms and other relevant clinical data were obtained through the clinical record and the questionnaire included in the Supplementary Data (S1 and S2 Tables).

**Fig 2:**
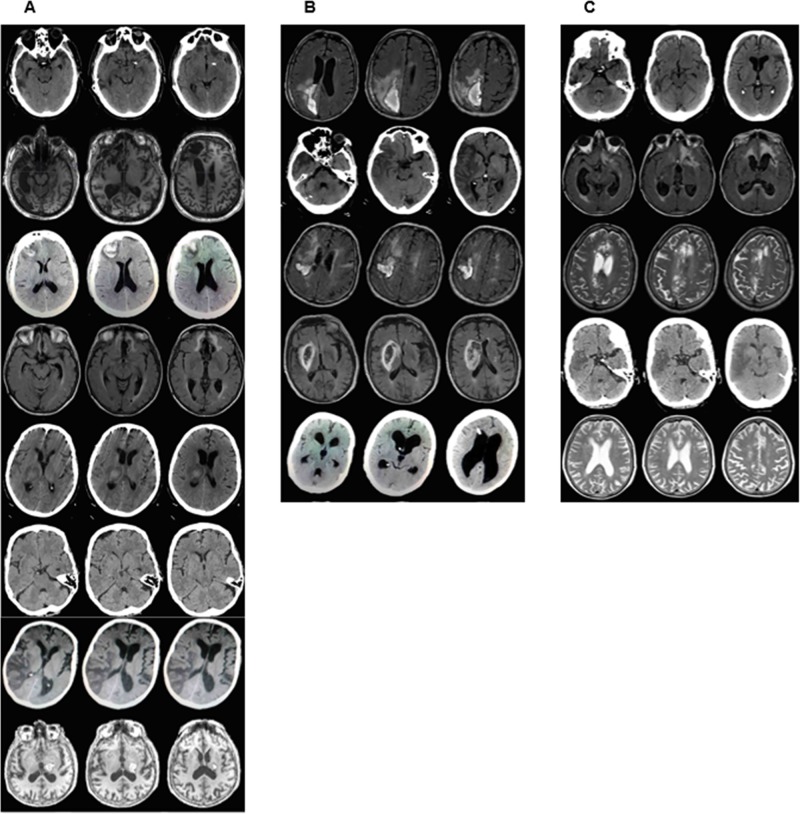
Neuroimaging data. Magnetic resonance imaging (MRI) and computerized tomography (CT) of each patient showing the main lesion: A) experimental group; B) 'pure' control group; C) 'mixed' control group. The images of two patients in the experimental group (subjects 7 and 10) are missing because it was not possible to access their medical records.

The first two patients in the experimental group had received neuropsychological rehabilitation before the present study, without any improvement in their confabulations. The remaining participants had not undergone any cognitive or behavioral interventions before the present study, since they had been hospitalized to receive physiotherapy. As described in Table 1 and shown in Fig 2, although with different etiologies, all the patients had lesions either in their frontal areas or in their right hemisphere regions. The higher number of patients with right hemisphere lesions is consistent with the increased frequency of confabulations, paramnesic misidentification, and anosognosia in these patients [3,28]; it is also consistent with the presence of severe language disorders in patients with homologous lesions in the left hemisphere. Table 1 shows patients’ demographic data and information on their lesions, as well as the distribution of the patients between the experimental and control groups. The CT and MRI images of most patients are shown in Fig 2.

**Table 1:** Patient demographic and lesion data.

Group	Age	Gender	Education (in years)	Etiology of lesion	Time from lesion (in months)	Neuro-image	Localization
**Exp.**	58	M	8	TBI	12	CT	Bifrontal and right temporal contusions
	35	M	12	TBI	60	MRI	Right fronto-temporal hematoma
	68	F	8	TBI	4	CT	Right fronto-parietal subdural hematoma
	68	F	12	Aneurysm in ACoA	8	MRI	Frontobasal bilateral
	62	M	8	Stroke in rMCA	4	CT	Right thalamus-capsular hemorrhage
	86	F	12	Stroke in rMCA	4	MRI	Right parietal hematoma
	59	M	8	Stroke in rMCA	5	-	Basal ganglia and right frontal lacunar infarcts
	78	M	8	Stroke in rMCA	3	CT	Right MCA territory ischemia
	73	M	8	Stroke in rMCA	3	MRI	Right thalamocapsular hematoma
	52	M	6	CO2 intoxication	4	-	Diffuse
**Total Exp.**	mean = 63	3F/7M	mean = 9		mean = 10.7		
	s.d. = 14.3		s.d. = 2.16		s.d. = 17.5		
**Pure Control**	51	M	12	Stroke in rMCA	5	MRI	Right occipito-parietal hematoma
	80	F	6	Stroke in rMCA	6	CT	Right MCA territory ischemia
	74	M	8	Stroke in rMCA	4	MRI	Right basal ganglia ischemia
	64	M	8	Stroke in rMCA	3	CT	Right frontal hemorrhage
	69	F	6	Hydrocephalus	42	CT	Diffuse
**Mixed Control**	74	M	6	TBI	5	CT	Bifrontal subdural hemorrhage
	66	F	15	Aneurysm in ACoA	10	MRI	Frontobasal bilateral
	48	M	12	Aneurysm in ACoA	5	MRI	Frontobasal bilateral
	81	F	8	Stroke in rMCA	3	CT	Right MCA territory ischemia
	74	F	6	Vascular MCI	12	MRI	Diffuse & right frontal hyperostosis
**Total Control**	mean = 68.1	5F/5M	mean = 8.7		mean = 9.5		
	s.d. = 11.2		s.d. = 3.19		s.d. = 11.8		

Exp. = experimental; s.d. = standard deviation; M = male; F = female; TBI = traumatic brain injury; ACoA = anterior communicating artery; rMCA = right middle cerebral artery; MCI = mild cognitive impairment; CT = computed tomography; MRI = magnetic resonance imaging.

The study was approved by the ethics committee of Virgen de las Nieves University Hospital (Granada, Spain) and the research met the ethical standards of the Declaration of Helsinki. Written informed consent was obtained from all the participants. A surrogate consent procedure was administered in the cases in which patients had a compromised capacity to consent. In those cases, a next of kin or legally authorized representative consented on behalf of the participants. This consent procedure was also approved by the same ethics committee (see S2 and S3 Files for more detailed information).

### Experimental treatment

#### Procedure

The experimental treatment was inspired by classical neuropsychological memory treatments. Participants had to learn some brief material (12 stimuli per session), after which they were asked for immediate and delayed recall. Each patient in the experimental group underwent a total of nine treatment sessions that were different from each other during three weeks, three sessions a week.

Regarding the learning phase, participants were told that they had to learn some stimuli and that they would have to answer questions about such stimuli later. The treatment sessions differed in the *type of material* that was presented, which was inspired by the classification of the Dalla Barba interview. Specifically, the classification was: 1) semantic memory for words and pictures; 2) general semantic memory; and 3) personal memory. There were 3 sessions of each type—nine sessions in total—and the order of presentation was counterbalanced between the sessions.

Concerning the *modality of the stimuli*, each session included both verbal and visual stimuli. The ‘words and pictures’ and ‘general semantic’ sessions also included stimuli to be imagined by the patient. The ‘personal’ sessions did not include imagined stimuli due to the difficulty of contrasting this information with reality, which we believe is critical for the treatment to be effective. The order of presentation of the stimuli was also counterbalanced across sessions. All the visual stimuli, including the words and texts to be read by the patient, were presented on the same 15-inch screen laptop computer. The therapist presented the rest of the stimuli (i.e., the words, texts, and stimuli to imagine).

Regarding the *nature of the source*, in each session the source was the patients themselves in half of the stimuli (i.e., they imagined objects, read pieces of news, or saw pictures of events of their lives) and the therapist in the other half (i.e., the therapist showed pictures of objects, read a text, or showed pictures of herself to the patients). The order of presentation of the stimuli was also counterbalanced across sessions. After the recall of the material, patients were asked to remember which modality corresponded to each recall (i.e., seen, heard, or imagined), and who had presented the material or who the information was about during the learning session (i.e., the therapist or themselves). Examples of the three variables—type of material, modality of the stimuli, and source—are shown in more detail in Table 2. More detailed descriptions of the stimuli are provided below, in the Stimuli section.

**Table 2:** Stimuli used in the treatment. Examples of stimuli organized depending on the type of material, the modality of stimuli, and the source (or subject providing the information).

Type of material	Modality of material	Source	Example of stimuli
Words and pictures	Visual	Therapist	Pictures of objects presented by the therapist (e.g., a guitar)
	Imagined	Patient	Objects imagined by the patient based on semantic categories (e.g., *Imagine an animal*)
	Verbal	Therapist	Words read by the therapist (e.g., *child*)
		Patient	Words read by the patient (e.g., *coffee*)
General semantic	Visual	Therapist	Faces of celebrities presented by the therapist (e.g., the king of Spain)
	Imagined	Patient	Celebrities imagined by the patient based on semantic categories (e.g., *Imagine an actor*)
	Verbal	Therapist	Brief news read by the therapist (e.g., *Enrique Morente died in Madrid*)
		Patient	Brief news read by the patient (e.g., *The first leg transplant in the world will take place in Valencia*)
Personal	Visual	Therapist	Photographs of the therapist (e.g., the therapist on the beach)
		Patient	Photographs of the patient (e.g., the patient at a wedding)
	Verbal	Therapist	Sentences about the therapist’s or the patient’s life events, read by the therapist (e.g., *Monica*—the therapist—*will go to the theater at the weekend*)
		Patient	Sentences about the therapist’s or the patient’s life events, read by the patient (e.g., *Monica went swimming yesterday*)

After the learning phase, the recall phase took place. In this phase, participants had to perform an immediate and a delayed recall test (after 10 minutes). A ‘free report’ paradigm—as opposed to recognition paradigms—was chosen because only this condition allows subjects to be responsible for the information that they have just produced [19]. At both moments (i.e., immediate and delayed), the patients were first asked for a free recall; if they could not remember the material, they were subsequently asked for a cued recall. We used the categories and the salient information of the stimuli (see the Stimuli section below for further details) as cues. Next, the patients were asked to attribute a source to each recollection. The therapist wrote the verbatim responses in a specially designed register where she could note all the answers and cues provided. Non-responses were permitted.

After both the immediate and the delayed recall, the participants were confronted with feedback about their correct responses, non-responses, and errors (i.e., confabulations and errors of attribution). The main feature of this feedback was that errors were verified in 100% of the cases, without giving patients the benefit of the doubt. This allowed us to ‘break’ the anosognosia and made patients responsible for their errors. Importantly, the feedback was accompanied by the previously stimuli presented, which were shown again so that participants could trust the feedback and have no doubts about it, but without a new learning phase. Specific instructions were given that emphasized the need for patients to pay more attention to stimuli and be more careful before answering (i.e., 'Think before answering. If you do not remember, not answering is better than guessing').

Before and after the treatment, three sessions (one with each type of material) were administered without feedback. These three sessions were identical before and after the treatment, but used different material from that used in the nine treatment sessions to avoid practice effects.

#### Stimuli

In the ‘semantic memory for words and pictures’ session, the patients read or listened to words, watched pictures of objects, or imagined objects. Words were extracted from a Spanish database of four-letter words [29] in which the words were organized by frequency (per 5 million words) and orthographic neighborhood. The selected words did not have any neighbors with greater frequency than themselves and had F5 frequency, that is, they appeared more than 100 times per 5 million words. We excluded all the words that were not nouns (i.e., verbs, prepositions, adverbs, and adjectives) and all those that could be both a noun and another category (e.g., the word ‘swim’ can be a noun or a verb). Pictures were extracted from a Spanish picture database [30] in which images are organized by semantic categories and classified by familiarity on a scale of 0 to 5, with 5 being the highest familiarity. Images with a familiarity score greater than 3.5 and belonging to different semantic categories were selected. The same categories were used for imagined stimuli (e.g., the patient was asked to imagine a fruit).

In the ‘general semantic memory’ session, the patients read or listened to news pieces, watched pictures of celebrities, or imagined celebrities. The news events were selected from a local newspaper, due to the absence of a database of this type of material. News pieces were selected based on six categories: politics, current news, tabloid press, health, sports, and bullfighting/culture. Half of the stimuli contained news about celebrities and the other half contained news about current local or national events. None had a length greater than 100 ± 10 words and all included a headline with the most relevant information. The pictures of celebrities were extracted from a Spanish database of famous faces (Espinosa & Arnedo, unpublished) in which faces are organized by semantic categories and classified by familiarity (on a scale of 0 to 5, with 5 being the highest familiarity) and by the percentage of success in naming them. Faces with a familiarity greater than 3.5 and success in naming >80% were selected. The same categories were used for imagined stimuli.

Finally, in the ‘personal memory’ session, the patients were exposed to pictures of themselves or of the therapist as well as sentences about daily events involving them or the therapist. The pictures of patients and biographical information were provided in advance by the relatives. Half of the sentences referred to past events while the other half referred to immediate or future activities. A maximum of four people appeared in each photograph. Half of the photos depicted specific autobiographical events with a reference stimulus (e.g., a wedding or a trip) and the other half depicted events without any specific reference (e.g., a beach day or time spent in a bar with friends). A maximum of four people appeared in each photograph. Both the semantic categories and the episodic references of each sentence and photograph were used in the cued recall.

### Neuropsychological assessment

#### Procedure

Both groups of patients underwent a neuropsychological assessment before the treatment to identify the similarities them in neuropsychological variables other than the confabulations. The same assessment was administered after the treatment to assess the possible effect of the treatment on other cognitive processes and to record possible undesired effects. The five patients of the 'mixed' control group (who subsequently received the treatment) were assessed before the *waiting list* time and after the treatment. They were not assessed after the *waiting list* time to avoid the practice effect in some tests. As a result, the five patients of the 'pure' control group only received the first assessment.

#### Stimuli

As mentioned above, we administered the Dalla Barba Provoked Confabulation Interview to confirm the presence of momentary confabulations. We also performed the Auditory A Test to exclude patients with alertness deficits. This test is a *sustained attention* task in which patients have to identify when the therapist says the letter 'a', among other different letters. In addition, we administered two tasks (visual extinction and line cancellation) to detect the presence of *neglect syndrome*, as 10 patients had experienced a stroke in the right middle cerebral artery. We evaluated *selective attention* to study its relationship with early deficits leading to confabulations [31] using the Picture Completion subtest of the Wechsler Adult Intelligent Scale, 3^rd^ edition (WAIS-III). In this task, subjects have to identify the missing part of a picture.

We also assessed variables that had been highlighted in previous studies. Specifically, we included *memory* tests to assess learning, recall, and recognition abilities, since these patients are usually amnesic and show multiple intrusions in recall as well as false positives in recognition [4,32]. Regarding auditory memory, we administered the Spanish version of the California Verbal Learning Test (i.e., *Test de Aprendizaje Verbal España Complutense*, TAVEC). The test has a learning phase (5 trials to learn a list of 16 words); an interference phase during which a new list is presented once; a recall phase with both short- and long-term recall, as well as free and cued recall; and finally a recognition phase, during which individuals have to recognize the 16 words between different distractors. We assessed visual memory with the Rey Complex Figure Test, in which subjects copy a figure. After a brief delay, they have to draw what they remember.

Regarding *executive functions*, we included an animal fluency test, which has been associated with the controlled evocation deficit shown by confabulators [5–6,13]. We also included measures of working memory, with the Digits subtest of the WAIS-III for the verbal component and the Spatial Location subtest of the Wechsler Memory Scale, 3^rd^ edition (WMS-III) for the visuo-spatial component. Abstract reasoning was assessed with the Similarities subtest of the WAIS-III, in which patients are asked about the similarity between two words. Planning was assessed with the Key Search subtest of the Behavioral Assessment of Disexecutive Syndrome (BADS), in which patients have to plan a route to find a lost object (the house key) in a large space (a field). We did not use tests that could be contaminated by the presence of neglect in patients, such as the Wisconsin Card Sorting Test or Stroop tasks.

### Measurements

The primary outcome measures were the sum of *confabulations*, *correct responses*, or *non-responses*. These measures were recorded during the three sessions without feedback before and after the treatment. The confabulations recorded were 1) guessed answers, 2) confusions in time and space, 3) a mixture of two or more stimuli presented, and 4) devised or bizarre responses. The scores ranged from 0 (no confabulations) to an unlimited number because of devised or bizarre responses. Regarding the correct responses and non-responses, the scores ranged from 0 (no correct answers) to 72 (12 stimuli remembered twice in each session: in the immediate and delayed recall).

The secondary outcome measures were the sum of the *errors* and *correct responses in source attribution*. The scores ranged from 0 (if all the answers were non-responses) to an unlimited number (depending on the number of confabulations produced by patients).

### Statistical analysis

The IBM SPSS 23 package was used to analyze the normality assumption for the experimental and control groups as well as for the 'mixed' control group by performing a Shapiro-Wilk test. All the groups were normally distributed as regards their primary and secondary outcome measures (all *ps*> 0.070). However, the assumption of normality was not met in several demographic and neuropsychological variables.

Next, in order to assess the effectiveness of the treatment, we tested for significant differences between the experimental and control groups by comparing the first two time-point measures (pre- and post-treatment vs. pre- and post-waiting). Mean scores in *confabulations*, *correct responses*, and *non-responses*, as well as *correct source attribution* and *errors in source attribution* were submitted to a 2 (Group: experimental vs. control) x 2 (Time-point: pre- vs. post-) mixed analysis of variance (ANOVA), with the first variable as a between-participants factor and the other as a within-participants variable. Next, a dependent samples Student’s *t* test was used to specifically analyze each group. First, we analyzed the improvement observed in the experimental group and also obtained Cohen's *d* to examine the effect sizes (small 0.2, medium 0.5, and large 0.8) [33]. Second, we compared each variable at the three point-times (pre-waiting, post-waiting, and post-treatment) of the 'mixed' control group. The long-term measurements were not analyzed due to the small number of patients in each condition (3, 6 or 18 months after the treatment finished).

Finally, we performed a nonparametric Mann-Whitney *U* test for independent samples to compare the demographic data and the neuropsychological assessment in both the experimental and control groups before treatment to confirm that they did not differ in any of the variables. Meanwhile, we performed a Wilcoxon test for paired samples to compare the assessment before and after treatment. This involved combining the experimental and the 'mixed' control groups (i.e., 15 patients) to explore the effect of the treatment on other cognitive processes.

## Results

The groups did not differ in age, education, or time elapsed since the injury (all *ps*> 0.405).

### Experimental treatment results

A mixed 2 (Group) x 2 (Time-point) ANOVA was performed on the mean scores of *confabulations*, *correct responses*, and *non-responses*, as well as those of *correct source attribution* and *errors in source attribution* (detailed data are presented in Table 3). Note that pre- and post-waiting measures were analyzed in the control group, while pre- and post-treatment measures were analyzed in the experimental group, through planned comparisons.

**Table 3:** Scores before and after the treatment. Mean scores and standard deviations (sd) for confabulations, correct responses, and non-responses and for both correct responses and errors in source attribution, organized by group and time-point measure. Note that the post-treatment measurement in the control group was performed by 5 patients. Note also that the direct scores of a single patient are shown in the post-treatment follow-up measure after both 9 and 18 months.

GROUP	TIME-POINT (Sample)		RESPONSES				
			Confabula-tions	Correct responses	Non-responses	Correct source attributions	Errors in source attribution
**Control group**	**Pre-waiting (N = 10)**	Mean (sd)	29.9 (13.8)	20.9 (10.6)	29.5 (11.8)	25.0 (13.2)	25.7 (12.5)
	**Post-waiting (N = 10)**	Mean (sd)	35.5 (11.3)	22.0 (8.9)	19.2 (8.8)	26.7 (12.1)	30.2 (11.6)
	**Post-treatment (N = 5)**	Mean (sd)	11.0 (7.4)	39.2 (16.4)	22.6 (11.3)	37.6 (10.3)	12.4 (4.0)
**Exp. group**	**Pre-treatment (N = 10)**	Mean (sd)	30.0 (9.9)	22.0 (13.6)	26.3 (15.8)	26.1 (13.7)	25.1 (6.1)
	**Post-treatment (N = 10)**	Mean (sd)	8.1 (4.2)	42.6 (18.7)	22.3 (17.5)	38.0 (15.6)	12.2 (7.0)
	**Post-treatment after 3 months (N = 3)**	Mean (sd)	14.7 (13.5)	36.0 (18.4)	23.0 (7.9)	35.0 (20.1)	14.3 (14.9)
	**Post-treatment after 9 months (N = 1)**	Direct score	0	50	22	49	1
	**Post-treatment after 18 months (N = 1)**	Direct score	6	59	12	50	13

**Fig 3:**
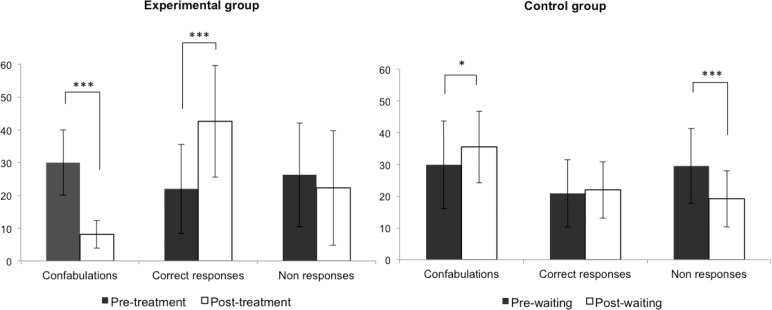
Main results for both the experimental (left) and control (right) groups. When we compared the pre-treatment and post-treatment measures, the experimental group showed a significant reduction in confabulations and an increase in correct responses, but non-responses remained the same. However, when the pre-waiting and post-waiting measures were compared, confabulations not only did not decrease but even significantly increased; non-responses decreased, while correct responses remained the same.

Regarding **confabulations**, the predicted Group x Time-point interaction was observed, *F*(1,18) = 64.99; *p*<0.0001; μ^2^ = 0.783. The groups did not differ in the pre-measures, *F*<1, but differed significantly in the post-measures, *F*(1,18) = 51.60; *p*<0.0001. Specifically, as can be observed in Fig 3, the experimental group showed a significant decrease in the number of confabulations after the treatment, *F*(1,18) = 82.43; *p*<0.0001, whereas the control group not only did not improve but even showed more confabulations after three weeks without any intervention, *F*(1,18) = 5.39; *p* = 0.032.

In the case of **correct responses**, the Group x Time-point interaction was also significant, *F*(1,18) = 20.55; *p* = 0.0002; μ^2^ = 0.533. Again, the groups did not differ in the pre-measures, *F*<1, but differed in the post-measures, *F*(1,18) = 9.91; *p* = 0.005. The experimental group showed a significant increase in the number of correct responses after the treatment, *F*(1,18) = 45.87; *p*<0.0001, while the correct responses did not change in the control group, *F*<1.

Concerning **non-responses**, the main effect of Time-point was significant, *F*(1,18) = 22.55; *p* = 0.0001; μ^2^ = 0.556, whereas the Group x Time-point interaction was marginally significant, *F*(1,18) = 4.38; *p* = 0.051; μ^2^ = 0.195. In this case, the groups did not differ in either the pre- or post-measures, both *Fs*<1. Specifically, the change in non-responses did not reach statistical significance in the experimental group after the treatment, *F*(1,18) = 3.53; *p* = 0.077, which suggests that patients did not improve on the basis of a non-responding strategy. However, non-responses decreased in the control group after the three weeks without treatment, *F*(1,18) = 23.40; *p* = 0.0001. This result, together with the increase in confabulations, seems to suggest that the control group became worse with the mere passage of time (Fig 3).

Finally, regarding **correct source attribution**, the main effect of Time-point was significant, *F*(1,18) = 7.10; *p* = 0.016; μ^2^ = 0.283, and the Group x Time-point interaction was marginally significant, *F*(1,18) = 4.00; *p* = 0.060; μ^2^ = 0.181. In fact, as shown by planned comparisons, the performance of the control group did not differ between the time-point measurements, *F*<1, while the experimental group showed a significant increase in correct source attribution, *F*(1,18) = 10.88; *p* = 0.004. When we analyzed the **errors in source attribution**, the Group x Time-point interaction was significant, *F*(1,18) = 21.46; *p* = 0.0002; μ^2^ = 0.544. Planned comparisons showed that the performance of the control group did not differ between measurements, *F*(1,18) = 2.87; *p* = 0.107. However, the experimental group showed a significant decrease in the number of errors in source attribution, *F*(1,18) = 23.60; *p* = 0.0001.

The calculations of Student’s *t* test and Cohen's *d* for the experimental group confirmed the previous results: confabulations decreased significantly, *t*(9) *=* 7.59; *p*<0.0001, with a huge effect size, *d* = 2.85. Correct responses increased significantly, *t*(9) *=* -5.39; *p* = 0.0004, also showing a large effect size, *d =* 1.25. However, non-responses did not change, *t*(9) *=* 1.51; *p* = 0.164 and *d =* 0.24.

More specifically, fewer **confabulations** were observed after treatment independently of the *type of material* (words and pictures, general semantic, or personal), the *modality of the stimuli* (visual, verbal, or imagined), the *moment of the recall* (immediate or delayed), and the *type of recall* (free or cued), all *ps*<0.002. An increase in **correct responses** was also observed in all these conditions (all *ps*<0.023), with the exception of the free recall condition, for which the difference was marginal, *t*(9) *=* -2.15; *p* = 0.060. **Non-responses** decreased marginally for the imagined stimuli, *t*(9) *=* 2.21; *p* = 0.054, but no significant changes were found in the number of non-responses for any other variable (all *ps*>0.191). Regarding **source attribution**, the number of correct source attribution responses increased significantly after treatment, *t*(9) *=* -2.71; *p* = 0.024, while the number of errors in source attribution decreased significantly, *t*(9) *=* 6.83; *p*<0.0001.

**Fig 4:**
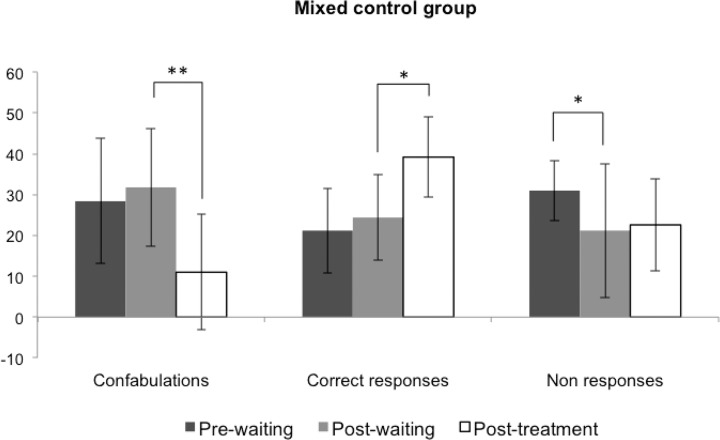
Main results of the treatment in the ‘mixed’ control group. Confabulations decreased and correct responses increased only after the treatment was administered.

Finally, a dependent samples Student's *t* test was performed to compare each variable at the three time points (i.e., pre-waiting, post-waiting, and post-treatment) of the 'mixed' control group. As in the experimental group, the number of **confabulations** decreased significantly only after the treatment, *t*(4) = 4.73; *p* = 0.009, and an increase in **correct responses** was only observed after the intervention, *t*(4) = -3.85; *p* = 0.018. As shown before, **non-responses** decreased between the pre- and post-waiting measures, *t*(4) = 4.36; *p* = 0.012, but remained the same after the treatment, as in the experimental group, *t*(4) = -1.16; *p* = 0.311 (see Fig 4). Regarding **source attribution**, again the number of errors only decreased after the treatment, *t*(4) = 3.82; *p* = 0.019, whereas the increment of hits was marginally significant, *t*(4) = -2.33; *p* = 0.080.

It is important to note that the changes observed after treatment were generalized to patients’ everyday lives as reported by family members (e.g., fewer arguments and less supervision in activities of daily living). Likewise, after treatment, behaviorally spontaneous and fantastic confabulations, reduplicative paramnesia, Fregoli syndrome, and pseudohallucinations had decreased significantly or fully disappeared (see Table 4). Finally, the effect of treatment persisted beyond 18 months. Detailed data are included in Table 3.

**Table 4:** Confabulations shown by patients and other comorbid symptoms. Mnestic and non-mnestic confabulations reported by relatives and therapists at pre-treatment or pre-waiting (Pre), post-waiting (Post-W), and post-treatment (Post-T).

		Behaviorally spontaneous confabulations			Fantastic confabulations			Fregoli syndrome			Reduplicative paramnesia			Pseudohallucinations		
Group	Etiology	Pre	Post-W	Post-T	Pre	Post-W	Post-T	Pre	Post-W	Post-T	Pre	Post-W	Post-T	Pre	Post-W	Post-T
**Exp. group**	TBI	Daily	-	Absent	Absent	-	Absent	Daily	-	Absent	Daily	-	Absent	Sporadic	-	Absent
	TBI	Daily	-	Absent	Absent	-	Absent	Sporadic	-	Absent	Absent	-	Absent	Absent	-	Absent
	TBI	Daily	-	Absent	Sporadic	-	Absent	Daily	-	Absent	Absent	-	Absent	Daily	-	Absent
	ACoA	Daily	-	Absent	Daily	-	Absent	Daily	-	Absent	Daily	-	Absent	Daily	-	Absent
	rMCA*	Daily	-	Absent	Daily	-	Absent	Daily	-	Absent	Daily	-	Absent	Daily	-	Absent
	rMCA*	Daily	-	Absent	Absent	-	Absent	Sporadic	-	Absent	Absent	-	Absent	Sporadic	-	Absent
	rMCA	Sporadic	-	Absent	Sporadic	-	Sporadic	Daily	-	Sporadic	Daily	-	Absent	Daily	-	Sporadic
	rMCA	Daily	-	Absent	Sporadic	-	Absent	Daily	-	Absent	Absent	-	Absent	Daily	-	Absent
	rMCA*	Daily	-	Sporadic	Daily	-	Sporadic	Daily	-	Absent	Daily	-	Absent	Daily	-	Absent
	CO2 Intoxication	Daily	-	Absent	Absent	-	Absent	Sporadic	-	Absent	Absent	-	Absent	Sporadic	-	Absent
**Total experimental group**		10/10	-	1/10	6/10	-	2/10	10/10	-	1/10	5/10	-	0/10	9/10	-	1/10
**Pure control group**	rMCA*	Daily	Daily	-	Daily	Daily	-	Daily	Daily	-	Daily	Daily	-	Daily	Daily	-
	rMCA*	Daily	Daily	-	Daily	Daily	-	Daily	Daily	-	Daily	Daily	-	Absent	Absent	-
	rMCA*	Daily	Daily	-	Sporadic	Sporadic	-	Daily	Daily	-	Daily	Daily	-	Daily	Daily	-
	rMCA*	Daily	Daily	-	Absent	Absent	-	Daily	Daily	-	Daily	Daily	-	Daily	Daily	-
	Hydroce-phalus	Daily	Daily	-	Absent	Absent	-	Daily	Daily	-	Daily	Daily	-	Daily	Daily	-
**Mixed control group**	TBI	Daily	Daily	Absent	Daily	Daily	Absent	Daily	Daily	Sporadic	Daily	Daily	Absent	Daily	Daily	Absent
	ACoA	Daily	Daily	Sporadic	Absent	Absent	Absent	Daily	Daily	Daily	Daily	Daily	Absent	Daily	Daily	Sporadic
	ACoA	Daily	Daily	Absent	Absent	Absent	Absent	Daily	Daily	Sporadic	Daily	Daily	Absent	Daily	Daily	Absent
	rMCA*	Daily	Daily	Absent	Sporadic	Sporadic	Absent	Daily	Daily	Absent	Daily	Daily	Absent	Daily	Daily	Absent
	Vascular MCI	Daily	Daily	Absent	Sporadic	Sporadic	Absent	Sporadic	Sporadic	Absent	Sporadic	Sporadic	Absent	Absent	Absent	Absent
**Total control group**		**10/10**	**10/10**	**1/5**	**6/10**	**6/10**	**0/5**	**10/10**	**10/10**	**3/5**	**10/10**	**10/10**	**0/5**	**8/10**	**8/10**	**1/5**
**Total**		**20/20**	**10/10**	**2/15**	**12/20**	**6/10**	**2/15**	**20/20**	**10/10**	**4/15**	**15/20**	**10/10**	**0/15**	**17/20**	**8/10**	**2/15**

Exp. = experimental; TBI = traumatic brain injury; AcoA = anterior communicating artery; rMCA = right middle cerebral artery; MCI = mild cognitive impairment; Daily = once or more per day; Sporadic = once or less per week; Absent = not present.

* Patients with neglect syndrome.

### Neuropsychological results

Before the treatment, the Mann-Whitney *U* test for independent samples did not show any differences between the experimental and the control group in any of the neuropsychological variables assessed (all *ps*>0.126). In addition, differences between the 'pure' and 'mixed' control groups were not significant (all *ps*>0.095). See Table 5.

**Table 5:** Neuropsychological results before the treatment. Mean scores and standard deviations (in parentheses) for each test in both control (pre-waiting) and experimental (pre-treatment) groups, as well as the results of the comparison between them. The scores of the control group are divided into two subgroups ('pure' and 'mixed').

FUNCTION	Pre-Waiting			Pre-Treat.	Comparison
*Test*, *subtest*, *and score*	Pure	Mixed	Total	Exp.	Mann-Whitney *U*
	N = 5	N = 5	N = 10	N = 10	
**Confabulations**					
Dalla Barba Provoked Confabulation Interview					
*Total number of confabulations*	20.0 (6.7)*	15.4 (4.3)*	17.7 (5.8)*	18.4 (3.6)*	*U* = 46.5; *p* = 0.790
**Attention**					
Sustained attention. Continuous Performance Test					
*Auditory A Test (Total errors)*	0.4 (0.5)	0.4 (0.5)	0.4 (0.5)	2.0 (3.3)	*U* = 39.0; *p* = 0.355
Selective attention					
*Picture Completion Subtest of WAIS-III (SS)*	3.2 (2.5)*	5.2 (1.8)*	4.2 (2.3)*	4.4 (4.4)*	*U* = 41.5; *p* = 0.512
**Verbal memory**					
Test Aprendizaje Verbal España Complutense TAVEC** (DS)					
*Learning*	19.6 (6.7)*	21.8 (8.1)*	20.7 (7.1)*	21.7 (10.9)*	*U* = 39.5: *p* = 0.964
*Short-term free recall*	1.4 (1.7)*	0.8 (1.3)*	1.1 (1.4)*	2.1 (2.0)*	*U* = 27.5; *p* = 0.249
*Short-term cued recall*	2.6 (2.5)*	2.4 (2.3)*	2.5 (2.3)*	3.0 (2.1)*	*U* = 33.5; *p* = 0.559
*Long-term free recall*	0.6 (0.9)*	1.0 (2.2)*	0.8 (1.6)*	2.4 (3.1)*	*U* = 26.0; *p* = 0.172
*Long-term cued recall*	2.8 (1.8)*	1.4 (1.1)*	2.1 (1.6)*	2.3 (1.9)*	*U* = 36.0; *p* = 0.709
*Intrusions in free recall*	4.6 (2.1)	7.6 (3.8)*	6.1 (3.3)*	14.4 (20.4)*	*U* = 30.5: *p* = 0.397
*Intrusions in cued recall*	8.8 (2.3)*	10.8 (7.3)*	9.8 (5.2)*	14.0 (12.9)*	*U* = 32.5; *p* = 0.503
*Perseverations*	2.4 (4.8)	3.2 (2.7)	2.8 (3.7)	7.1 (8.3)	*U* = 24.5; *p* = 0.163
*Recognition*	11.6 (3.8)*	13.0 (2.3)*	12.3 (3.1)*	12.4 (3.1)*	*U* = 37.5; *p* = 0.822
*False positives in recognition*	8.2 (4.5)*	14.6 (5.0)*	11.4 (5.6)*	11.1 (6.2)*	*U* = 39.0; *p* = 0.929
*Discriminability index*	71.6 (4.6)*	60.0 (14.9)*	65.8 (12.1)*	64.7 (10.3)*	*U* = 32.0; *p* = 0.476
**Visual memory**					
Rey Complex Figure Test					
*Immediate recall (DS)*	1.0 (0.0)*	1.0 (0.0)*	1.0 (0.0)*	2.0 (2.9)*	*U* = 35.0; *p* = 0.126
*Presence of confabulations in immediate recall (no*. *of patients with confabulations/total patients)* ***	3/5	3/5	6/10	7/10	*p* = 0.645
**Constructive praxis**					
Rey Complex Figure Test					
*Copy (DS)*	1.0 (0.0)*	6.6 (5.9)*	3.8 (2.9)*	2.9 (3.2)*	*U* = 42.0; *p* = 0.752
*Presence of confabulations in copy (no*. *of patients with confabulations/total patients)* ***	0/5	0/5	0/10	0/10	*p* = 1.000
**Executive functions**					
Digit Span Subtest of WAIS-III (SS)	8.0 (2.1)	9.6 (3.0)	8.8 (2.6)	7.6 (3.5)*	*U* = 40.0; *p* = 0.447
Spatial Span Subtest of WMS-III (SS)	5.2 (2.9)*	6.8 (1.3)*	6.0 (2.3)*	6.1 (2.5)*	*U* = 48.5; *p* = 0.909
Similarities Subtest of WAIS-III (SS)	9.2 (2.4)	7.6 (4.5)*	8.4 (3.5)	9.2 (4.4)	*U* = 49.0; *p* = 0.939
Animal Fluency Test (DS)	6.8 (2.6) *	8.8 (3.7)*	7.8 (3.2)*	8.7 (4.5)*	*U* = 48.5; *p* = 0.909
Planning-Key Search Test of BADS (DS)	3.6 (2.7)*	5.8 (5.5)*	4.7 (4.2)*	5.5 (3.6)*	*U* = 35.5; *p* = 0.433

WAIS-III = Wechsler Adult Intelligence Scale, 3^rd^ edition; WMS-III = Wechsler Memory Scale, 3^rd^ edition; BADS = Behavioral Assessment of Disexecutive Syndrome; DS = direct score; SS = scaled score.

* Pathological scores

** Spanish version of the California Verbal Auditory Test

*** The proportion of confabulations was analyzed using a two-tailed chi-square test with 95% confidence.

Patients were also assessed after treatment, which allowed us to observe the effects of treatment on other neuropsychological variables as well as the presence of undesired consequences. A Wilcoxon test for paired samples (see Table 6) showed a significant reduction of confabulations in the Dalla Barba interview (*T* = 3.0; p = 0.001) and a significant improvement in some of the variables related to confabulations in previous literature.

**Table 6:** Neuropsychological results after the treatment. Mean scores and standard deviations (in parentheses) for each test, both at pre-treatment and post-treatment, and results of the comparison between them. Only data from 15 patients are reported (10 from the experimental group and 5 from the 'mixed' control group).

FUNCTION	Pre-T	Post-T	Wilcoxon Test
*Test*, *subtest*, *and score*	N = 15	N = 15	
**Confabulations**			
Dalla Barba Provoked Confabulation interview			
*Total number of confabulations*	17.4 (3.9)*	7.4 (4.3)	***T* = 3.0; *p* = 0.001**
**Attention**			
Sustained attention. Continuous Performance Test			
*Auditory A Test (Total errors)*	1.5 (2.7)	0.3 (0.7)	*T* = 5.0; *p* = 0.249
Selective attention			
*Picture Completion Subtest of WAIS-III (SS)*	4.7 (3.7)*	6.4 (2.1)*	*T* = 24.5; *p* = 0.079
**Verbal memory**			
Test Aprendizaje Verbal España Complutense, TAVEC** (DS)			
*Learning*	21.8 (9.5)*	34.7 (30.2)*	*T* = 34.0; *p* = 0.421
*Short-term free recall*	1.6 (1.8)*	1.9 (2.8)*	*T* = 26.0; *p* = 0.878
*Short-term cued recall*	2.8 (2.1)*	3.3 (2.8)*	*T* = 21.5; *p* = 0.540
*Long-term free recall*	1.9 (2.8)*	2.1 (2.4)*	*T* = 13.0; *p* = 0.484
*Long-term cued recall*	2.0 (1.7)*	3.1 (2.9)*	*T* = 6.0; *p* = 0.176
*Intrusions in free recall*	11.8(16.1)*	5.4 (10.2)	***T* = 0.0; *p* = 0.003**
*Intrusions in cued recall*	12.8 (10.8)*	3.9 (3.9)	***T* = 5.0; *p* = 0.013**
*Perseverations*	5.6 (6.8)	2.6 (3.1)	***T* = 4.0; *p* = 0.028**
*Recognition*	12.6 (2.8)*	13.2 (3.4)*	*T* = 32.5; *p* = 0.964
*False positives in recognition*	12.5 (5.8)*	7.4 (5.6)	***T* = 7.0; *p* = 0.012**
*Discriminability index*	62.9 (11.9)*	76.7 (9.7)	***T* = 6.5; *p* = 0.010**
**Visual memory**			
Rey Complex Figure Test			
*Immediate recall (DS)*	1.7 (2.4)*	6.6 (10.8)*	*T* = 1.0; *p* = 0.079
*Presence of confabulations in immediate recall* *(no*. *of patients with confabulations/total patients)* ***	11/15	4/15	***p* = 0.015**
**Constructive praxia**			
Rey Complex Figure Test			
*Copy (DS)*	7.1 (12.3)*	22.3 (29.9)*	*T* = 7.0; *p* = 0.066
*Presence of confabulations in copy (no*. *of patients with confabulations/total patients)* ***	0/15	0/15	*p* = 1.000
**Executive functions**			
Digit Span Subtest of WAIS-III (SS)	8.3 (3.4)	8.7 (2.3)	*T* = 32.0; *p* = 0.583
Spatial Span Subtest of WMS-III (SS)	6.3 (2.2)*	7.2 (1.9)*	*T* = 8.0; *p* = 0.085
Similarities Subtest of WAIS-III (SS)	8.7 (4.4)	10.1 (2.3)	*T* = 21.0; *p* = 0.286
Animal Fluency Test (DS)	8.7 (4.1)*	9.8 (5.1)*	*T* = 35.5; *p* = 0.485
Planning-Key Search Test of BADS (DS)	5.6 (4.1)*	7.8 (3.3)	*T* = 18.0; *p* = 0.055

WAIS-III = Wechsler Adult Intelligence Scale, 3^rd^ edition; WMS-III = Wechsler Memory Scale, 3^rd^ edition; BADS = Behavioral Assessment of Disexecutive Syndrome; DS = direct score; SS = scaled score.

* Pathological scores

** Spanish version of the California Verbal Auditory Test

*** The proportion of confabulations was analyzed using a two-tailed chi-square test with 95% confidence.

More specifically, *selective attention* improved but not significantly (picture completion: *T* = 24.5; *p* = 0.079). Regarding the *memory variables*, we found significant reductions in perseverations (*T =* 4.0; *p* = 0.028) and intrusions in free and cued recall (*T* = 1.0; *p* = 0.003 and *T* = 5.0; *p* = 0.013, respectively), and a significant decrease in false positives in recognition (*T* = 7.0; *p* = 0.012), with a consequent improvement in the ability to discriminate between learned and novel material (*T* = 6.5; *p* = 0.010)—note that the discriminability index is computed by comparing hits and false positives in recognition—. However, none of the other memory variables changed after treatment and the deficits in learning and recall persisted (all *ps>*0.176). Moreover, although deficits in immediate recall and copy of the Rey Complex Figure improved, they persisted (*T* = 1.0; *p* = 0.079 and *T* = 7.0; *p* = 0.066, respectively), but the presence of confabulations (i.e., houses or rockets) decreased significantly (*p* = 0.015; a chi-square test was conducted with a 95% confidence interval to compare the proportion of confabulations between performance at pre-treatment and post-treatment). With regard to *executive functions*, none of the variables changed after the treatment, although planning showed a marginal improvement (key search: *T =* 18.0; *p* = 0.055). Therefore, since the neuropsychological variables improved or remained the same, administering the treatment did not lead to any undesired effects.

## Discussion

This study presents the effectiveness of a new treatment for confabulations after an acquired brain injury. To our knowledge, this is the first time that a treatment of this nature has been administered to a group of confabulators and tested against a *waiting list* control group. Patients only improved after the treatment was administered, as observed in both the experimental and the 'mixed' control groups. After treatment, patients were not only able to remember the information more correctly but also to determine the modality of their recall (i.e., visual, verbal, or imagined) and the source of the information (i.e., the therapist or themselves). Moreover, this improvement was reflected in other neuropsychological variables and in the daily lives of patients according to their relatives' reports, and seemed to persist over time. Importantly, all patients improved, regardless of the etiology of their confabulations.

The current results are promising, although some limitations should be acknowledged in the study, due to the small sample and the potential bias caused by conducting an interim analysis before recruiting more patients to complete the sample. Future studies should replicate the effectiveness of the present study in a larger sample of patients and compare the results of the treatment group against those observed in an active control group; this will make it possible to achieve a more appropriate blinding procedure. Also, it will be interesting for future studies to verify whether a similar improvement occurs in other types of confabulations associated with progressive brain injury, such as Alzheimer’s disease, or in patients with disorders caused by alcohol abuse, such as Korsakoff syndrome. It would also be interesting to know whether patients showing spontaneous but not momentary confabulations also benefit from our treatment.

Importantly, our results cannot be explained on the basis of spontaneous recovery, as shown by the second assessment of the *waiting list* control group. Indeed, our patients had been confabulating for several months without any improvement, as demonstrated by the reports of their family members. In fact, the second measure taken after three weeks (equivalent to the duration of the treatment) showed an increase in confabulations in the control group as well as a decrease in non-responses. Therefore, letting patients confabulate seems to induce more confabulations rather than spontaneous recovery. It is important to note that in real life it is usually difficult, if not impossible, to show confabulators definitive evidence demonstrating their confabulations. This is because patients usually make secondary supporting claims that may be simple rationalizations or secondary confabulations [34] and sometimes even their confabulations have some real evidence. Another important factor why family confrontation is usually not effective could be that patients might not trust relatives as much as they trust the therapist, who has some medical authority over them that relatives do not have. All these factors may explain why confrontation by the therapist in an experimentally controlled and systematic setting clearly seems to work, whereas non-systematic confrontation by relatives sometimes even increases confabulations.

Furthermore, our treatment has some innovative traits compared to those implemented previously. First, it managed to decrease the number of confabulations in a very short time, so it can be easily applied in hospitals allowing for early intervention. Second, it focuses only on confabulations without applying other therapies that may interfere with the results, and uses simple and versatile materials that can be adapted to the age, educational level, and sensorimotor difficulties of each patient. Finally, it is based on a variety of tasks influencing multiple processes that are traditionally impaired in confabulators and always implies the active participation of the patient, which is considered a very effective method in rehabilitation [10].

Besides the relevance of these results from a clinical point of view, they may also have theoretical implications for explanatory models of confabulations. Why was the treatment so effective after only nine sessions? Which processes are involved in such an improvement? Confabulations clearly cannot be explained by a memory deficit alone because some amnesic patients do not confabulate. In addition, the patients whose confabulations decreased with our treatment still showed general deficits in verbal and visual memory, although treatment decreased perseverations, intrusions in free recall, and false positives in recognition.

In addition to memory problems, deficits in executive functions have also been proposed, particularly in the monitoring and top-down control processes involved in memory retrieval [19]. Nevertheless, some authors consider instead that bottom-up mechanisms are the basis of confabulations. For example, Ciaramelli et al. [6] consider the main deficit of confabulators to be early processing of irrelevant information, while Schnider [12] attributes confabulations to an impairment in reality filtering. Perhaps both top-down and bottom-up mechanisms are involved [19,35–36]. The tasks included in this treatment are evidently associated not only to top-down mechanisms (e.g., awareness of memory problems and the falsehood of some memories, reorientation regarding the temporal context, monitoring, and logical inferences) but also to bottom-up mechanisms (e.g., careful visual search, familiarity, and global and local perception). Indeed, the joint action on both top-down and bottom-up mechanisms may be responsible for the efficacy of our treatment in such short time.

In any case, if all these processes were impaired directly by the brain injury, their restoration would be impossible or would take longer than three weeks. The fast recovery can only be explained if some of these processes were affected but not completely impaired after brain damage. As proposed by Ciaramelli et al. [6], top-down processes may be preserved but not recruited. As proposed by Shallice [34,37], this strategic system is recruited 1) when competing stimuli are likely to produce a mistake; 2) in conditions of uncertainty; or 3) when online monitoring is required. Our results suggest that in confabulators this early competition between representations and the consequent uncertainty never happens by default, leading to a selection of representations that the system does not verify and consequently does not call into question. In fact, confabulators usually have no doubts about their memory (this is known as the *feeling of rightness*). However, and importantly, the patients in our study reduced their outputs in the post-treatment measure, expressed low certainty in the few confabulations that appeared sporadically after treatment, and were more aware of their memory deficits. Therefore, the treatment seems to restore these memory control processes, a result recently reported in patients who had a history of confabulations, compared to those who still confabulated [38]. Whether this improvement in control processes is what makes patients gain insight or vice versa should be tested in future studies.

Recently, Gosh and Gilboa [39] reviewed the characteristics of memory schemas. Cross-connectivity is an important feature to which schemas are sensitive, with the consequent competition between them in the selection process (see [40–43] for other models suggesting cumulative mechanisms in a successful retrieval). Therefore, a schema will be selected when its activation is greater than that of its competitors. Confabulations could be related to schemas that are sensitive to cross-connectivity, and failure in the competition processes may be the key factor in producing them. Based on these models, we suggest that the selection of a specific representation in memory (either a visual representation of a real object or a representation of an autobiographical, past event) depends on early processing of the properties of the stimuli. As the properties of a stimulus are being processed, certain representations are activated and others are discarded. Activated representations start to accumulate matched properties. A representation will be selected if it shows the highest correspondence with the properties of the target stimulus (e.g., an object, a face, or an episodic event). Consequently, error detection and verification processes will not be recruited because they are not necessary. By contrast, if several (i.e., two or more) representations achieve an equivalent accumulation of matched properties, the monitoring processes will be recruited to detect the conflict and begin an effortful and strategic search to resolve it, adding information that will be used to select one representation over its competitors.

In fact, we have observed that confabulators showed serious difficulties in a visual search task, especially when the target and the distractors were physically similar and therefore had interconnected representations in memory [44]. In addition, they also seemed to have difficulties detecting a stimulus-response conflict (i.e., Simon effect), especially when the target was surrounded by distractors [45]. Future research should determine whether confabulations are related to altered processing of stimuli properties or to conflict detection deficits.

Such a mechanism may explain the similarities between mnestic and non-mnestic confabulations. These two types of confabulations are supported by different neural circuits: the anterior limbic system in mnestic confabulations and right hemispheric lesions in non-mnestic confabulations, which could explain the dissociation often described in the literature [3,46]. However, these neural bases seem to have a common target in the ventromedial prefrontal cortex which may account for the frequency of comorbidities, the similarities between them, and the improvement of both after our treatment. Future studies exploring the common and different neural bases as well as cognitive mechanisms involved in mnestic and non-mnestic confabulations could be of great interest to better understand this phenomenon.

Finally, changes in the neuropsychological tasks also provided some clues about what may happen after treatment. Normal scores in working memory and similarities usually indicate that the dorsolateral circuits involved in executive functions are preserved in most confabulators [5, 47]. However, we observed a large number of intrusions and false positives in memory tasks and an impairment of selective attention in picture completion and planning. In the Picture Completion subtest (WAIS-III), any detail could catch the patient’s attention and prevent him/her from tracking the entire figure to find the missing part. For example, almost all of the patients in our sample were unable to realize the absence of a leg on a table or the absence of a nose on a face. Erroneously, they thought that a tablecloth or a vase was missing from the table, and glasses or a hat were missing from a face. Moreover, when presented with the Rey Complex Figure, confabulators focused their attention on specific details without attending holistically to the figure and thereafter developed a response based on these details with absolute certainty, as mentioned above. For example, they drew houses, tents, or mills attending to the triangular shape on the right, drew stairs attending to the four lines on the left, or drew a face attending to the circle with three dots inside.

After treatment, the greatest improvements were observed in several discriminability scores, with results never described in the literature before. Specifically, perseverations, false positives, and intrusions were reduced significantly, as were the number of confabulations in the Rey Complex Figure Test. Although these results should be interpreted with caution, they seem to support the idea that the top-down ability to monitor and selectively attend to stimuli properties and control the output improved after treatment. Since several of our patients showed both mnestic and non-mnestic confabulations, it will clearly be interesting to perform future correlation and regression analyses with a larger sample size to study which neuropsychological variables are related to each type of confabulation in similar or differential ways.

In summary, this study presents a useful and efficient tool in the clinical setting that could improve the quality of life of confabulators, as it dramatically reduced their confabulations in just three weeks, after nine sessions of treatment. Future studies should replicate the results of the treatment in a larger sample. Although assessing the genesis of confabulations was not the purpose of our study, we also obtained some clues about the mechanisms involved in generating confabulations, pointing to a deficit in the early selection of interconnected representations in memory and a preservation of top-down retrieval-monitoring processes, which are nevertheless not used during confabulations. The empirical basis for selective attention and conflict-detection deficits in selecting representations from memory should also be tested in future studies. Finally, it would be interesting to introduce event-related potentials (ERPs) or functional neuroimaging studies before and after the treatment. This would allow testing whether the treatment leads to a functional recruitment of monitoring processes that depend on more lateral circuits, while early deficits—which presumably depend on more ventromedial and anterior limbic circuits—remain impaired. Moreover, studies involving diffusion tensor imaging could help to define the circuits involved in different types of confabulations in order to better understand the common and differing mechanisms. The fact that changes in white matter have been detected with these techniques after training [48] makes such future studies quite promising.

## Supporting information

S1 Table Clinical record. The therapist completed it for each patient based on both the medical history and the neuropsychological assessment.
(DOCX)

S2 Table Confabulations (and related symptoms) questionnaire administered to relatives before and after the treatment. The questions were asked by the therapist who also wrote down the answers.
(DOCX)

S1 File CONSORT 2010 checklist. This checklist includes all the information to include when reporting a randomised trial.
(PDF)

S2 File Spanish version of the memory of the project. The memory was submitted to the Ethics Committee of Virgen de las Nieves University Hospital (Granada, Spain) for its approval.
(PDF)

S3 File English translation of the memory of the project submitted to the Ethics Committee of Virgen de las Nieves University Hospital (Granada, Spain). (PDF)
