# Supplementary material for: Effectiveness of a neuropsychological treatment for confabulations after brain injury: A clinical trial with theoretical implications
Source: PLoS One. 2017 Mar 3;12(3):e0173166. doi: 10.1371/journal.pone.0173166 (PMC5336256; doi:10.1371/journal.pone.0173166)
Supplement: S1 Table — The therapist completed it for each patient based on both the medical history and the neuropsychological assessment. (DOCX) [file pone.0173166.s001.docx]

**S1 Table**

| PERSONAL INFORMATION   - Birth date: - Gender: - Civil status: - Years of education: - Occupation: |
| --- |
| MEDICAL HISTORY   - Neurodegenerative disease: - Acquired brain injury (TBI, stroke, etc.): - Psychiatric disorders (psychosis, depression, anxiety, etc.): - Alcohol or drugs: - Normal or corrected vision: - Normal or corrected audition: - Other diseases: - Current medication: |
| CURRENT ILLNESS   - Reason for admission: - Date of the lesion: - Neuroimage report (CT, MRI…): - Other relevant medical information (surgeries, epilepsy, etc.): |
| COGNITIVE DEFICITS RELATED TO CONFABULATIONS   - Orientation:   - Time:   - Space:   - Person: - Sleep-wake cycle: - Motor agitation: - Anosognosia: - Changes in personality:   - Irritability:   - Aggressiveness:   - Impulsivity:   - Rigidity:   - Delay intolerance:   - Frustration intolerance: - Behavior changes   - Verbal disinhibition:   - Behavior disinhibition: - Memory:   - Memory for events of the recent past:   - Retention of new information: - Attention (sustained, selective, and divided): - Language (aphasia or other language/speech disorders): |
