# Supplementary material for: Effectiveness of a neuropsychological treatment for confabulations after brain injury: A clinical trial with theoretical implications
Source: PLoS One. 2017 Mar 3;12(3):e0173166. doi: 10.1371/journal.pone.0173166 (PMC5336256; doi:10.1371/journal.pone.0173166)
Supplement: S2 Table — The questions were asked by the therapist who also wrote down the answers. (DOCX) [file pone.0173166.s002.docx]

**S2 Table**

| **Behaviors** | **Never** | **Sporadically (Once or less per week)** | **Frequent (2 to 6 times per week)** | **Daily (Once or more per day)** |
| --- | --- | --- | --- | --- |
| **Spatio-temporal confusion of real events** |  |  |  |  |
| Question (Q): Does the patient change the moment or the place where the events actually happened? (e.g., he/she claims that he/she travelled last week, but this happened several weeks, months or years ago). How often? |  |  |  |  |
| **Presence of false memories** |  |  |  |  |
| Q: Does the patient talk about events that did not occur? (e.g., he/she claims that he/she has been in the swimming-pool, but never was). How often? |  |  |  |  |
| **Behavior according to false memories** |  |  |  |  |
| Q: When the patient produces a false memory, does he/she try to act on it? (e.g., he/she wants to go to work or shopping). How often? |  |  |  |  |
| **Fantastic and unbelievable histories**  Q: Does the patient talk about unbelievable histories that are impossible? (e.g., he/she claims to have flown or to have seen animals in unsuitable places). How often? |  |  |  |  |
| **Hard to convince (frequent arguments)** |  |  |  |  |
| Q: Do you have difficulties convincing him/her about his/her mistakes? Are the arguments frequent for this reason? How often? |  |  |  |  |
| **Pseudohallucinations** |  |  |  |  |
| Q: Does the patient confuse the objects he/she sees to the point that he/she seems to have hallucinations? (e.g., he/she sees a monkey sitting on a chair when, in fact, it is a backpack). How often? |  |  |  |  |
| **Fregoli syndrome** |  |  |  |  |
| Q: Has the patient a hyperfamiliarity with strangers? Does he/she recognize strangers as if they were familiar people? How often? |  |  |  |  |
| **Reduplicative paramnesia** |  |  |  |  |
| Q: Does the patient duplicate places or people? How often? |  |  |  |  |
| **Confabulations impact** |  |  |  |  |
| Q: How do these changes impact on the social, work, and family environment? How often does the patient need supervision? |  |  |  |  |
