# Supplementary material for: Effectiveness of a neuropsychological treatment for confabulations after brain injury: A clinical trial with theoretical implications
Source: PLoS One. 2017 Mar 3;12(3):e0173166. doi: 10.1371/journal.pone.0173166 (PMC5336256; doi:10.1371/journal.pone.0173166)
Supplement: S2 File — The memory was submitted to the Ethics Committee of Virgen de las Nieves University Hospital (Granada, Spain) for its approval. (PDF) [file pone.0173166.s004.pdf]

HOSPITAL SAN RAFAEL
ORDEN HOSPITALARIA DE SAN JUAN DE DIOS

ugr Universidad de Granada

Hospital Universitario Virgen de las Nieves
Servicio Andaluz de Salud
CONSEJERÍA DE SALUD

MEMORIA DEL PROYECTO

ESTUDIO DE LOS CIRCUITOS NEUROANATÓMICOS, VARIABLES PREDICTORAS Y FACTORES DE PRONÓSTICO DE LA CONFABULACIÓN ESPONTÁNEA: DISEÑO DE UN PROGRAMA DE EVALUACIÓN Y REHABILITACIÓN

INTRODUCCIÓN

Los pacientes con daño en estructuras del encéfalo basal tales como el córtex ventromedial y el sistema límbico anterior (cuerpos mamilares, núcleos anteriores del tálamo, corteza perirrinal, parte inferior del cíngulo anterior, etc.) suelen mostrar confabulaciones (Turner et al., 2010). Se trata de memorias falsas que el paciente cree que son verdaderas y que genera sin intención de engañar al oyente, con tal grado de certeza que se aferra a ellas a pesar de las evidencias en contra llegando a actuar en consonancia con ellas —*behavioraly confabulations*—, con el consiguiente impacto funcional en la vida diaria del paciente y de la familia y la necesidad de supervisión continua. En general, es considerada como una patología grave, persistente, poco habitual y de mal pronóstico (Malloy, Bihrle, Duffy, y Cimino, 1993).

La confabulación se ha relacionado tradicionalmente con el síndrome de Korsakoff, en el que el consumo abusivo de alcohol y el déficit de tiamina afecta estructural y funcionalmente al sistema límbico anterior (cuerpos mamilares y tracto mamilo-talámico). Sin embargo, existe una condición clínica que también se asocia frecuentemente a las confabulaciones y en la que las variables relacionadas con el consumo de sustancias y la desnutrición no se hallan presentes: la hemorragia subaracnoidea (HSA) por rotura de los aneurismas de la Arteria Comunicante Anterior (ACoA) que dan lugar al denominado *Síndrome de ACoA* que incluye cambios de personalidad, amnesia y confabulaciones. Asimismo, las confabulaciones se pueden producir tras daño cerebral traumático, accidentes cerebro-vasculares, encefalitis o hidrocefalia (Turner et al., 2010).

Existen diversas aproximaciones teóricas y una literatura relativamente extensa acerca de los procesos implicados en la confabulación espontánea. En concreto, las distintas hipótesis apuntan a déficits en los procesos de recuperación de memoria (p.e., ver Gilboa, 2010; Johnson et al., 2002; Schnider et al., 2006). Sin embargo, no hay prácticamente ningún estudio centrado en conocer las variables predictoras o los factores de pronóstico funcional, ni en aplicar estos conocimientos para la correcta evaluación y la rehabilitación más efectiva de estos pacientes, cuyas confabulaciones, como hemos mencionado, interfieren de manera significativa con su vida diaria. De hecho, las confabulaciones (mucho más que las alteraciones de memoria o en funciones ejecutivas) les hacen totalmente dependientes de la supervisión de sus familiares y/o del entorno para las actividades de la vida diaria (AVD).

Basándonos en dicha literatura sobre las confabulaciones, el Servicio de Neuropsicología del Hospital Universitario San Rafael de Granada en colaboración con el Grupo de Neurociencia de la Universidad de Granada, ha diseñado un protocolo de evaluación y un tratamiento experimental consistente en una combinación de estrategias de rehabilitación neuropsicológicas. En concreto, consiste en un tratamiento tradicional de memoria donde los

pacientes deben aprenderse 12 estímulos (palabras, imágenes, caras, noticias o fotos) y recordarlos de forma inmediata y demorada. Tras la fase de recuerdo, se les proporciona feedback acerca de las confabulaciones cometidas, así como de las respuestas correctas y las no respuestas, insistiendo en que presten atención a los detalles (entrenamiento en atención selectiva), que no respondan de forma precipitada (entrenamiento en inhibición de respuestas irrelevantes) y que comprueben su respuesta (entrenamiento en detección de error).

OBJETIVOS DE LA INVESTIGACIÓN

Los objetivos de la presente investigación, por tanto, son:

1. **Estudiar las variables predictoras de la confabulación** en una población de pacientes que han sufrido daño cerebral. Para ello se establecerán las variables que puedan predecir la aparición de confabulaciones como secuela (antecedentes personales; etiología y forma de presentación del daño cerebral; tipo de tratamiento; complicaciones). Esto permitirá avanzar en el estudio de las estructuras cerebrales implicadas en este cuadro neuropsicológico, así como definir qué pacientes tienen más probabilidades de presentar confabulaciones.

2. **Estudiar los resultados obtenidos con el tratamiento experimental**, aplicándolo a un grupo de pacientes así como a un grupo control de no confabuladores (pacientes con daño cerebral equivalente sin confabulaciones). Esto nos permitirá también estudiar las diferencias entre ambos tipos de pacientes (confabuladores y no confabuladores) en cuanto a todas las variables predictoras comentadas en apartados anteriores.

3. **Seguir la evolución de los pacientes** que han recibido el tratamiento a medio y largo plazo, comparándola con la de aquellos pacientes sin tratamiento (pacientes confabuladores que no accedan al tratamiento por propia decisión). Esto permitirá observar la efectividad del tratamiento a largo plazo, así como estudiar variables de buen y mal pronóstico funcional.

4. **Estudiar los procesos cognitivos asociados**. Las hipótesis actuales apuntan a déficits en los procesos de recuperación de memoria. Nuestra hipótesis de partida es la presencia de un déficit temprano en procesos de atención selectiva, que preceden a los problemas de recuperación. En este sentido, ya se han diseñado varias tareas experimentales para comprobar dicha hipótesis: tareas de enmascaramiento de estímulos y tareas de búsqueda selectiva entre distractores. Esto nos permitirá avanzar en el conocimiento de los mecanismos cognitivos implicados para detectar mejor a estos pacientes, evaluarlos de forma adecuada y mejorar el tratamiento para que sea lo más eficiente posible.

REQUISITOS DE LA INVESTIGACIÓN

Participantes

Criterios de inclusión: pacientes que hayan sufrido daño cerebral y que hayan ingresado en el Servicio de Neurocirugía del *Hospital Universitario Virgen de las Nieves* o en el Servicio de Medicina Interna del *Hospital Universitario San Rafael*. Se entrevistará a la familia y/o al paciente para valorar la presencia de confabulaciones mediante una entrevista breve y sencilla (ver Anexo 1). Se explicará al paciente y a la familia la posibilidad de participar en el estudio, se les entregará la hoja informativa y el consentimiento informado (Anexos 2 y 3), de forma que se contactará con aquellos que deseen participar. En función de la presencia o no de confabulaciones, los pacientes, una vez obtenido su consentimiento, pasarán a formar parte del *grupo experimental* (confabuladores) o del *grupo control* (no confabuladores).

Criterios de exclusión: 1) pacientes cuyas confabulaciones estén asociadas a niveles de alerta disminuidos o a demencia; 2) pacientes intervenidos que además presenten otras alteraciones neurológicas asociadas, problemas de salud mental o abuso de drogas; 3) pacientes que rehúsen participar. En este último caso, a los pacientes confabuladores se les propondrá participar en un *grupo control sin tratamiento* (confabuladores sin tratamiento).

Variables de interés

Se registrarán variables de distinto tipo para poder llevar a cabo los objetivos propuestos:

1. Variables demográficas: edad, sexo, formación académica, etc.
2. Variables relacionadas con los antecedentes personales del paciente: factores de riesgo cardiovascular (antecedentes de hipertensión arterial, obesidad, diabetes, dislipemia), hábitos tóxicos (tabaquismo o alcoholismo), enfermedad cerebrovascular previa, etc.
3. Variables relacionadas con el tipo y el momento del tratamiento: médico y quirúrgico.
4. Variables relacionadas con complicaciones: ausencia o presencia de resangrado, de vasoespasmo (clínico y/o en imagen) o de lesión isquémica (clínico y/o en imagen), necesidad de drenaje ventricular externo o derivación de LCR definitiva. Otros factores relacionados con la morbilidad hospitalaria: infecciones, úlceras por decúbito, tromboembolismo, duración del ingreso, etc.
5. Variables relacionadas con el resultado: Glasgow Outcome Scale (GOS) y Rankin al alta.
6. Variables de neuroimagen: Resonancia Magnética
7. Variables comportamentales:
    a. puntuaciones en pruebas neuropsicológicas atencionales, de memoria y de funciones ejecutivas.

b. puntuaciones en tareas experimentales: principalmente, tiempos de reacción, falsas alarmas y omisiones.

c. puntuaciones en el tratamiento: número de confabulaciones, respuestas correctas, no respuestas y fallos de atribución en las líneas base pretratamiento y postratamiento.

Diseño y procedimiento

El estudio tendrá carácter prospectivo, para lo cual se aprovechará bien el ingreso inicial del paciente, bien el momento en el que el paciente acuda a la cita de revisión a los 6 o a los 12 meses. Mediante una breve entrevista a la familia (Anexo 1) se diferenciarán los pacientes confabuladores y los no confabuladores. Tras facilitar la información y obtener el consentimiento informado, se recogerán las variables demográficas, de lesión y neuroimagen. Estas variables se recabarán de la historia clínica del paciente. La neuroimagen se realizará en caso de que el paciente no tenga ninguna disponible anteriormente o bien que la que tenga no sea la más adecuada. Inicialmente se promoverá que, siempre que sea posible, las imágenes sean en Resonancia Magnética, adaptando esta preferencia a las posibilidades del paciente y de los Servicios de Radiología. A partir de ese momento, el procedimiento seguirá un diseño de caso único A-B-A, siendo A las líneas base y B el tratamiento.

- Línea base pretratamiento: evaluación neuropsicológica (que servirá también para excluir a pacientes con alteraciones atencionales o demencia) y realización de las tareas experimentales.
- Tratamiento: administración del tratamiento experimental.
- Línea base postratamiento: evaluación neuropsicológica y tareas experimentales postratamiento.

La duración total del estudio será aproximadamente de 3 meses para cada paciente. Los pacientes serán citados en el Hospital Universitario San Rafael con una frecuencia de 2-3 sesiones a la semana. Tras la finalización del estudio, se hará entrega de un informe neuropsicológico.

VOLUNTARIEDAD Y CONFIDENCIALIDAD

La participación en la investigación es totalmente voluntaria y los datos obtenidos serán siempre confidenciales según la*Ley 15/1999 de Protección de Datos de Carácter Personal*. A todo participante se le proporcionará una Hoja Informativa además de informarles verbalmente sobre la investigación, y firmarán un Consentimiento Informado (Anexos 2 y 3).

Además, los datos personales que se le requieren (como la edad, sexo, formación académica y datos de salud) son los necesarios para cubrir los objetivos del estudio. En ninguno de los informes del estudio aparecerá su nombre, y su identidad no será revelada a persona alguna salvo para cumplir con los fines de la investigación.

El acceso a dicha información queda restringido al personal autorizado que está obligado a mantener la confidencialidad de la información. Los resultados de la investigación podrán ser comunicados a las autoridades sanitarias y a la comunidad científica a través de congresos y/o publicaciones. Los datos podrán ser también utilizados con otros fines de carácter científico.

De acuerdo con la ley vigente, cada participante tiene derecho al acceso de sus datos personales; asimismo, y si está justificado, tiene derecho a su rectificación y cancelación. Por tanto, si el participante quiere abandonar la investigación podrá retirar su consentimiento cuando quiera, sin tener que justificar el por qué y sin que ello derive en ninguna consecuencia adversa para él. A partir de ese momento, sus datos serán retirados del estudio.

El tratamiento diseñado no supone ningún riesgo para la persona ya que sólo requiere respuestas motoras y verbales y, en todo caso, según otros tratamientos neuropsicológicos de memoria, esperamos que sea beneficioso para el paciente.

RESPONSABLES DE LA INVESTIGACIÓN

Servicio de Neuropsicología. Hospital Universitario San Rafael:
- Dra. Mónica Triviño Mosquera. Neuropsicóloga. Teléfono: 958 275700. e-mail: Monica.Trivino@sjd.es / mtrivino@ugr.es

Servicio de Neurocirugía. Hospital Universitario Virgen de las Nieves:
- Dr. Gonzalo Olivares Granados. Neurocirujano. Teléfono: 958 021553. E-mail: gonzalo.olivares.sspa@juntadeandalucia.es
- Dra. Ana Jorques Infante. Neurocirujana. Teléfono: 958 021553. E-mail:amjorques@gmail.com

Grupo de Neurociencia Cognitiva. Universidad de Granada:
- Dr. Juan Lupiañez Castillo. Catedrático de Psicología Experimental. Teléfono: 958 243766. E-mail: jlupiane@ugr.es
- Dra. Marisa Arnedo Montoro. Profesora Titular de Neuropsicología. Teléfono: 958 246268. E-mail: marnedo@ugr.es
- Estrella Ródenas García. Doctoranda. E-mail: estrelly_r@hotmail.com

ANEXO 1.

ENTREVISTA BREVE PARA LA DETECCIÓN DE CONFABULACIONES A FAMILIARES

1. ¿Está su familiar desorientado?
2. ¿Cambia de día sus recuerdos? Por ejemplo, cree haber hecho hoy algo que hizo hace días o semanas. O cree que algo que pasó hace años, ha sucedido en otro momento.
3. ¿Afirma que las cosas han sucedido de una forma que no es la correcta?
4. ¿Inventa cosas que no han pasado nunca? ¿Se cree lo que inventa?
5. ¿Tiene usted la sensación de que se ha vuelto "mentiroso"?
6. ¿Ve cosas que no están, como si tuviera alucinaciones?
7. ¿Confunde a las personas? ¿Dice conocer a gente desconocida? ¿Tiene sensación de familiaridad?
8. ¿Tienen discusiones frecuentes por estas "confusiones"? ¿Afirma lo que dice a pesar de demostrarle que no ha sido así?
9. ¿Quiere hacer cosas que no puede (como ir a trabajar, conducir, salir a la calle, etc.)? ¿Necesita supervisar todo lo que hace?
10. ¿Tiene problemas de memoria en general? ¿Es decir, es olvidadizo, no recuerda lo que ha hecho recientemente, etc.?
11. ¿Ha cambiado su forma de ser? ¿Está más desinhibido? ¿Irritable? ¿Agresivo? ¿Inorpotuno y descarado?
12. Si actualmente el paciente no se comporta así, ¿en algún momento llegó a comportarse así tras la intervención?

ANEXO 2.

Documento de información específica para otorgar el Consentimiento Informado

PROGRAMA DE INVESTIGACIÓN, EVALUACIÓN Y REHABILITACIÓN DE LAS CONFABULACIONES TRAS DAÑO CEREBRAL

Este documento se le ha entregado, porque usted o un familiar suyo ha sufrido una lesión cerebral. El motivo de darle esta información es invitarle a participar en un programa para atender las posibles secuelas que usted —o su familiar— puede sufrir tras dicho evento. Dicho programa es realizado por el Servicio de Neuropsicología del Hospital San Rafael de Granada, en colaboración con la Universidad de Granada y el Servicio de Neurocirugía del Hospital Universitario Virgen de las Nieves donde usted —o su familiar— se encuentra actualmente ingresado.

Tras una lesión cerebral, es posible que se produzcan una serie de secuelas. Las más comunes son:

- Secuelas cognitivas: problemas atencionales, pérdida de memoria reciente y confabulaciones. Las confabulaciones consisten en la producción de recuerdos falsos que el paciente cree que son verdaderos. El paciente no tiene intención de engañar y, en ocasiones, se aferra a su confabulación a pesar de las evidencias en contra.
- Secuelas conductuales: desinhibición motora y verbal. En ocasiones muestran cierta tendencia a no “estarse quietos” y hablan mucho, llegando a decir cosas inoportunas.
- Secuelas funcionales: dificultades para realizar algunas actividades de la vida diaria. Algunas veces tienen problemas con actividades más difíciles como cocinar o realizar compras, pero en otras ocasiones pueden tener dificultad para actividades más cotidianas como el aseo o el vestido.

Dichas secuelas pueden ser evaluadas y pueden mejorar con una rehabilitación cognitiva y funcional adecuada. Le invitamos a participar en nuestro programa, mediante el cual podremos investigar más en profundidad lo que sucede en estos pacientes, a la vez que evaluaremos y trataremos las secuelas que puedan estar presentes. Sin ningún coste por su parte. Lo único que necesitamos es que usted o su familiar deseen participar y colaborar con nosotros.

PROCEDIMIENTO

Si usted desea participar, únicamente nos debe proporcionar un teléfono de contacto. Nosotros nos encargaremos de contactar con ustedes y organizar los encuentros para realizar el programa.

El programa consistirá en lo siguiente:

- Evaluación neuropsicológica exhaustiva para evaluar las secuelas cognitivas, conductuales y funcionales. En caso de no presentar ninguna secuela, se le proporcionara un informe neuropsicológico.
- Realización de tareas experimentales: consisten en ejercicios de atención y de memoria que se realizan en un ordenador. Son tareas sencillas y fáciles de hacer, que nos proporcionan

información acerca de lo que sucede en estos pacientes. Eso nos permite diseñar mejor los tratamientos.

- En caso de presentar alguna de las secuelas, se llevarán a cabo los programas de intervención necesarios: intervención para las confabulaciones, para los problemas de atención y memoria, para los trastornos de conducta o para la realización de las actividades de la vida diaria. Tras las intervenciones se realizará una nueva evaluación y se le proporcionará un informe neuropsicológico.
- Finalmente, en el caso de ser necesario para la investigación, el programa también incluirá la realización de una Resonancia Magnética cerebral, sin ningún coste económico por su parte.

VOLUNTARIEDAD Y CONFIDENCIALIDAD

Este tipo de tratamientos no suponen ningún riesgo para la persona ya que sólo requieren respuestas verbales y motoras. Además, resultados con tratamientos neuropsicológicos de memoria similares muestran que son beneficiosos para el paciente. Es por ello, que le animamos a colaborar: con su participación nosotros podemos avanzar en nuestras investigaciones y, a cambio, usted o su familiar pueden recibir un servicio de evaluación y tratamiento gratuito (subvencionado por distintos proyectos de investigación del Ministerio de Educación y Ciencia, y de la Junta de Andalucía).

La participación en la investigación es totalmente voluntaria y los datos obtenidos serán siempre confidenciales según la *Ley 15/1999 de Protección de Datos de Carácter Personal*. Además, los datos personales que se le requieren (como la edad, sexo, formación académica y datos de salud) y la neuroimagen son los necesarios para cubrir los objetivos del estudio. En ninguno de los informes del estudio aparecerá su nombre, y su identidad no será revelada a persona alguna salvo para cumplir con los fines de la investigación. Cualquier información de carácter personal que pueda ser identificable será conservada y procesada por medios informáticos en condiciones de seguridad.

El acceso a dicha información queda restringido al personal autorizado que está obligado a mantener la confidencialidad de la información. Los resultados de la investigación podrán ser comunicados a las autoridades sanitarias y a la comunidad científica a través de congresos y/o publicaciones. Los datos podrán ser también utilizados con otros fines de carácter científico.

De acuerdo con la ley vigente, cada participante tiene derecho al acceso de sus datos personales; asimismo, y si está justificado, tiene derecho a su rectificación y cancelación. Por tanto, si el participante quiere abandonar la investigación podrá retirar su consentimiento cuando quiera, sin tener que justificar el porqué y sin que ello derive en ninguna consecuencia adversa para él. A partir de ese momento, sus datos serán retirados del estudio.

Si esta hoja informativa no es suficiente para el participante, siempre puede pedir información adicional sobre la investigación y el procedimiento.

Para contactar, le facilitamos los siguientes datos:

Dra. Mónica Triviño Mosquera
Servicio de Neuropsicología
Hospital Universitario San Rafael. Granada.
e-mail: Monica.Trivino@sjd.es
Teléfono: 958 275700

ANEXO 3

Documento de Consentimiento Informado

PROGRAMA DE INVESTIGACIÓN, EVALUACIÓN Y REHABILITACIÓN DE LAS CONFABULACIONES TRAS DAÑO CEREBRAL

Participante: ____

Persona de contacto: ____

Teléfono de contacto: _________________________

Persona que informa: ____

Este documento tiene como finalidad dejar constancia de que usted, o quien le represente, ha dado su consentimiento a participar en este programa de intervención y, por tanto, nos autoriza a recoger y utilizar sus datos tal y como se ha descrito en la hoja informativa. Antes de firmar este documento, usted debe haber sido informado de forma verbal y por escrito sobre la investigación.

CONSENTIMIENTO

Manifiesto que estoy conforme con la intervención y el protocolo que me han propuesto, y que he recibido y comprendido satisfactoriamente toda la información que considero necesaria para adoptar mi decisión. Asimismo, se me ha informado sobre mi derecho a retirar mi consentimiento en el momento que lo considere oportuno, sin obligación de justificar mi voluntad y sin que de ello se derive ninguna consecuencia adversa para mí, siendo retirados en ese momento mis datos del estudio. También manifiesto que se me ha informado sobre mi derecho a solicitar más información complementaria en el caso de que lo necesite.

Firma del participante<br><br><br>Fecha:	Firma persona representa<br><br>DNI:<br>Fecha:	Firma del informador:<br><br><br>Fecha:
Representación por:<br>□ Voluntad de la persona interesada<br>□ Minoría de edad<br>□ Incapacidad persona interesada	FIRMA POR REVOCACIÓN<br><br>Nombre:<br>DNI:<br>Fecha:	
