# Supplementary material for: Effectiveness of a neuropsychological treatment for confabulations after brain injury: A clinical trial with theoretical implications
Source: PLoS One. 2017 Mar 3;12(3):e0173166. doi: 10.1371/journal.pone.0173166 (PMC5336256; doi:10.1371/journal.pone.0173166)
Supplement: S3 File — (PDF) [file pone.0173166.s005.pdf]

MEMORY OF THE PROJECT

STUDY OF THE NEUROANATOMICAL CIRCUITS, PREDICTOR VARIABLES AND PROGNOSTIC FACTORS OF SPONTANEOUS CONFABULATION: DESIGN OF AN EVALUATION AND REHABILITATION PROGRAM

INTRODUCTION

Patients with damage in the basal brain structures such as the ventromedial cortex and anterior limbic system (mammillary bodies, anterior nucleus of the thalamus, perirhinal cortex, lower part of the anterior cingulum, etc.) usually show confabulations (Turner et al., 2010). Confabulations consist of the generation of false memories without intention to deceive. The patients believe that they are right, with such degree of certainty, that they cling to them despite the evidence against. Sometimes the confabulations even lead the patients to act in accordance with them —behaviorally confabulations— with the consequent functional impact on the daily life of the patient and relatives, since they need continuous supervision. Overall, it is considered a severe pathology, persistent, uncommon and with poor prognostic (Malloy, Bihrle, Duffy, y Cimino, 1993).

The confabulation has traditionally been related to Korsakoff's syndrome, where the abusive alcohol consumption and the thiamine deficiency affect structurally and functionally the anterior limbic system (mammillary bodies and mammilo-thalamic tract). However, it exists a clinical condition that is also frequently associated with confabulations and which the variables related to substance abuse and undernutrition are not present: the subarachnoid hemorrhage (SAH) after the rupture of the Anterior Communicating Artery (ACoA) aneurism, that is usually named as *ACoA Syndrome* which includes personality changes, amnesia and confabulations. Also confabulations may occur after traumatic brain injury, strokes, encephalitis or hydrocephalus, among others (Turner et al., 2010).

There are different theoretical approximations and a relatively extensive literature about the processes involved in spontaneous confabulation. Specifically, the different hypotheses point to deficits in memory retrieval processes (e.g., see Gilboa, 2010; Johnson et al., 2002; Schnider et al., 2006). However, there are practically no studies focused on either knowing the predictor variables or functional prognostic factors, nor on applying this knowledge for the correct assessment and the design of effective rehabilitation of these patients, whose confabulations, as we just mentioned, interfere significantly with their daily life. In fact, the confabulations (much more than the memory or executive functions deficits) the patients become on dependent persons who need the supervision of their relatives in the activities of daily living (ADL).

Based on the literature mentioned above, the Neuropsychology Service of the San Rafael University Hospital (Granada, Spain) —in collaboration with the Group of Cognitive Neuroscience at the University of Granada (Granada, Spain)— has designed an assessment protocol and an experimental treatment consisting of a combination of neuropsychological rehabilitation strategies. Specifically, it is a traditional memory treatment where patients must

learn 12 stimuli (words, images, faces, news or photos) and recall them immediately and after a delay of 10 minutes. After both recalls, a feedback is provided to patients informing them of the confabulations, as well as the correct answers and the no answers, insisting that they have to pay attention to details (selective attention training), they do not respond hastily (training in inhibition of irrelevant answers) and they need to check their responses (error detection training).

OBJECTIVES OF THE INVESTIGATION

The objectives of the present investigation, thus, are the following:

1. **Study the predictor variables of confabulation** in a population of patients who have suffered brain injury. We will study some of the variables that could predict the appearance of confabulations (medical history; etiology and form of presentation of brain injury; type and timing of treatment; complications). This would allow the advance of the study of the etiologies involved in this neuropsychological profile, as well as to define those patients who have more probabilities to present confabulations.
2. **Explore the results obtained with the experimental treatment**, applying it to a group of patients and a control group of non confabulators (patients with equivalent brain injury but without confabulations). This would also allow us to study the differences between both types of patients (confabulators and no confabulators) in all those predictor variables commented above.
3. **Follow up the evolution in long-term of patients** who have received treatment. This would allow us to observe the effectiveness of long-term effects of the treatment, as well as to study the variables related to good or poor functional prognostic.
4. **Study the associated cognitive processes.** The actual hypothesis points to deficits in memory retrieval processes. Our initial hypothesis is the presence of an early deficit in selective attention which precedes the retrieval problems. In this regard, we are designing several experimental tasks to check this hypothesis: a task with masked stimuli and a task of selective visual search. This would allow us to advance in the knowledge of the cognitive mechanisms involved in confabulations to better detect these patients, asses them and design a treatment as efficient as possible.

REQUIREMENTS OF THE INVESTIGATION

Participants

Inclusion criteria: confabulators who have suffered brain injury and are admitted to the Neurosurgery Service of the *Virgen de las Nieves University Hospital* or in the Internal

Medicine Service of the *San Rafael University Hospital* (both at Granada, Spain). The relatives and/or the patient will be interviewed to assess the presence of confabulations through a brief interview (see Annex 1). Then, we will explain to the patient and his/her relatives the possibility of participating in the study, and we will provide them the information sheet and the informed consent (Annexes 2 and 3), so we will contact with those who wish to participate. Once we obtained the informed consent, the patients will become part of the *experimental group* (confabulators) or the *control group* (no confabulators).

Exclusion criteria: 1) patients whose confabulations are associated with decreased levels of alertness or dementia; 2) patients who also have other neurological disorders, mental health problems or drug abuse; 3) patients who refuse to participate. In the latter case, the confabulators patients will be proposed to participate in a *no treatment control group* (confabulators without treatment).

Variables of interest

Variables of different types will be registered to carry out the proposed objectives:

1. Demographic variables: age, gender, academic formation, etc.
2. Variables related to the medical history of the patient: cardiovascular risk factors (arterial hypertension, obesity, diabetes, dyslipidemia), toxic habits (smoking or alcoholism), previous cerebrovascular disease, etc.
3. Variables related to the type and timing of treatment: medical and surgical.
4. Variables related to complications: absence or presence of hemorrhage, vasospasm (clinical and/or in neuroimage) or ischemic lesion (clinical and/or in neuroimage), need for external ventricular drainage or derivation of definitive CSF. Other factors related with hospital morbidity: infections, decubitus ulcers, thromboembolism, duration of hospital admission, etc.
5. Variables related to the result: Glasgow Outcome Scale (GOS) and Rankin at discharge.
6. Neuroimaging variables: Magnetic Resonance.
7. Behavioral variables:
    a. Scores on attentional neuropsychological tests, memory and executive functions.
    b. Scores on the treatment: number of confabulations, correct answers, no answers and attribution failures in the baselines pre-treatment and post-treatment.
    c. Scores on experimental tasks: principally, reaction times, false alarms and omissions.

The study will be prospective, so we will recruit the participants during the initial admission in the hospitals or when the patients come to review appointments at 6 or 12 months. Through a brief interview to the relatives (Annex 1), we will differentiate patients confabulators to those without confabulations. After the information phase and after obtaining the informed consents, we will collect demographic, medical and neuroimaging results. These variables will be collected from the patient's history. Neuroimaging will be made if the patient has not previously one available or if the image is not the most adequate. Initially it will be promoted, whenever it is possible, the images to be Magnetic Resonance, adapting this preference to the possibilities of the patient and the availability of the Radiology Services. From that moment, the procedure will follow a single case design A-B-A, being A the baselines and B the treatment.

- Baseline pre-treatment: neuropsychological assessment (it will also serve to exclude patients with attentional deficits or dementia) and administration of the experimental tasks.
- Treatment: administration of the experimental treatment.
- Baseline post-treatment: neuropsychological assessment and experimental tasks.

The total duration of the study will be approximately 3 months for each patient. Patients will be cited at the San Rafael University Hospital with a frequency of 2-3 sessions per week. After completion of the study, we will deliver a neuropsychological report.

WILLFULNESS AND CONFIDENTIALITY

The participation in this research is totally voluntary and the data obtained will always be confidential according to the Spanish *Law 15/1999 on Protection of Data of Personal Character*. All participants will be provided with an information sheet, verbally inform about the investigation, and will sign an informed consent (Annexes 2 and 3).

In addition, personal data required (as age, gender, academic formation and health data) are necessary to meet the objectives of the study. In none of the study reports will appear the participant names, and their identity will not be revealed to any person except to fulfill the objectives of the investigation.

Access to this information remains restricted to authorized personnel who are obliged to keep the confidentiality of the information. The research findings will be communicated to health authorities and the scientific community through conferences and/or publications. The data will not be used with no other purposes that the scientific.

According to the existing law, each participant has the right to access to his/her personal data; likewise, and if it is justified, they have the right to rectification and cancellation. Therefore, if the participant wants to abandon the investigation he/she will be able withdraw the consent when he/she wants, without having to justify the reasons and

without any adverse consequences derived from his/her decision. From that moment, the data will be removed from the study.

The designed treatment poses no risk to the person because it only requires motor and verbal responses and, in all case, according to similar neuropsychological memory treatments, we expect it to be beneficial to the patient.

RESPONSIBLE FOR INVESTIGATION

Service of Neuropsychology. San Rafael University Hospital:

- Dr. Mónica Triviño Mosquera. Neuropsychologist. Telephone: 958 275700. e-mail: Monica.Trivino@sjd.es / mtrivino@ugr.es

Service of Neurosurgery. Virgen de las Nieves University Hospital:

- Dr. Gonzalo Olivares Granados. Neurosurgeon. Telephone: 958 021553. E-mail: gonzalo.olivares.sspa@juntadeandalucia.es
- Dr. Ana Jorques Infante. Neurosurgeon. Telephone: 958 021553. E-mail: amjorques@gmail.com

Group of Cognitive Neuroscience. University of Granada:

- Dr. Juan Lupiañez Castillo. Professor of Experimental Psychology. Telephone: 958 243766. E-mail: jlupiane@ugr.es
- Dr. Marisa Arnedo Montoro. Professor of Neuropsychology. Telephone: 958 246268. E-mail: marnedo@ugr.es
- Estrella Ródenas García. PhD student. E-mail: estrelly_r@hotmail.com

ANNEX 1.

SHORT INTERVIEW FOR THE DETECTION OF CONFABULATIONS FOR RELATIVES

1. Is the patient disoriented?
2. Does the patient confound the moment when his/her memories occurred? For example, believing that he/she has done today something that made some days or weeks ago. Or believing that something that happened some years ago happened at another moment.
3. Does the patient affirm that things happened in a way that is not correct?
4. Does the patient invent things that have never happened? Does he/she believe what he/she invented?
5. Do you have the feeling that he/she has become a "liar"?
6. Does the patient see things that are distorted, as if he/she had hallucinations?
7. Does he/she confuse people? Does he/she "know" the unknown people? Does he/she have feelings of familiarity?
8. Do you usually have discussions with him/her because of those "confusions"? Does he/she reassert what he/she says despite it has been demonstrated it has not happened like that?
9. Does the patient want to do things he/she cannot do (as going to work, driving, going out to street, etc.)? Do you need to supervise everything he/she does?
10. Does the patient have memory problems in general? That is, is he/she forgetful, does he/she not remember what has done recently, etc.?
11. Has he/she changed the way of being? Is he/she more disinhibited / irritable / aggressive / inopportune /sassy than before?
12. If the patient does not currently display any of these behaviors, did he/she act like that at some moment after the brain injury?

HOSPITAL SAN RAFAEL
ORDEN HOSPITALARIA DE SAN JUAN DE DIOS

ugr Universidad de Granada

Hospital Universitario Virgen de las Nieves
Servicio Andaluz de Salud
CONSEJERÍA DE SALUD

ANNEX 2

Specific information document to give the Informed Consent

PROGRAM TO INVESTIGATION, EVALUATION AND REHABILITATION OF CONFABULATIONS AFTER BRAIN INJURY

The aim of this document is invite you to participate in a program to attend the possible sequels that you —or your relative— may have after suffering a brain damage. This program is conducted at the Neuropsychology Service of the *San Rafael University Hospital* (Granada, Spain), in collaboration with the University of Granada and the Neurosurgery Service of the *Virgen de las Nieves University Hospital* where you —or your relative— are now admitted.

After a brain damage, some sequels can happen. Some of them are:

- Cognitive sequels: attentional problems, recent memory loss or confabulations. The confabulations consist of the production of false memories that the patient believes that they are true. The patient has no intention to deceive and, at times, clings to his confabulation despite the evidence against.
- Behavioral sequels: motor and verbal disinhibition. Sometimes they show certain tendency to not "stand still" and talk a lot, saying ill-judged things.
- Functional sequels: difficulty performing some activities of daily living. Sometimes they have problems with activities like cooking or make purchases, but at other times they may have difficulty in easier everyday activities as grooming or dress.

These sequels can be evaluated and can improve with a proper cognitive and functional rehabilitation. We invite you to participate in our program, so we will be able to investigate more deeply what happens in these patients, at the same time that we will assess and treat the consequences that may be present. Without any cost on your part. The only thing that we need is that you or your relative wish to participate and cooperate with us.

PROCEDURE

If you wish to participate, solely you must provide us a contact telephone. We will handle to contact you and organize the encounters to make the program.

The program will consist of the next:

- Exhaustive neuropsychological evaluation to assess cognitive, behavioral and functional sequels. In case of no sequel at all, you will be provided with a neuropsychological report.
- Experimental tasks: consists of attention and memory exercises that are performed in a computer. They are easy and provide us information about what happens in these patients. This allows us to design better treatments.
- If necessary, the intervention program will take place: neuropsychological rehabilitation for the confabulations, for attention and memory problems, for behavioral disorders or for the realization of activities of daily living. Following the interventions we will perform a new evaluation and will provide you with a neuropsychological report.

- Finally and only if it is necessary for research, the program also include performing a magnetic resonance, without any economic cost on your part.

WILLFULNESS AND CONFIDENTIALITY

This type of treatments has no risk to the person since it only requires verbal and motor responses. Indeed, similar neuropsychological memory treatments are beneficial for the patients. That it is why we encourage you to collaborate: thanks to your participation we could advance in our investigations and, in exchange, you or your relative can receive a professional assessment and a free treatment (grantee by various investigation projects of the Ministry of Education and Science, and the Government of Andalucía, Spain).

The participation in the study is totally voluntary and the data obtained will always be confidential according to the Spanish Law 15/1999 on Protection of Data of Personal Character. In addition, personal data that are required (as age, gender, academic formation and health data) and the neuroimaging are necessary to meet the objectives of the study. In none of the reports of the study will appear your name, and your identity will not be revealed to any person except to fulfill the objectives of the investigation. Anyone information of personal character that may be identifiable will be conserved and processed by computerized means in safety conditions.

The access to such information will remain restricted to authorized personnel who is obliged to maintain the confidentiality of the information. The research findings will be able communicated to health authorities and the scientific community through conferences and/or publications. The data will not be used with other purposes than those of scientific character.

According to the existing law, each participant has the right to access to his/her personal data; likewise, and if it is justified, they also have the right to rectification and cancellation. Therefore, if the participant wants to abandon the investigation, he/she will be able to withdraw the consent when he/she wants, without having to justify the reasons and without any adverse consequences derived from it. Al that moment, the data will be removed from the study.

If this information sheet is not sufficient for you to understand the investigation, you can always ask additional information about the investigation and procedure.

To contact, we facilitate the following data:

Dr. Mónica Triviño Mosquera
Service of Neuropsychology
San Rafael University Hospital. Granada.
e-mail: Monica.Trivino@sjd.es
Telephone: 958 275700

HOSPITAL SAN RAFAEL
ORDEN HOSPITALARIA DE SAN JUAN DE DIOS

ugr Universidad de Granada

Hospital Universitario Virgen de las Nieves
Servicio Andaluz de Salud
CONSEJERÍA DE SALUD

ANNEX 3

Informed Consent Document

**PROGRAM FOR THE INVESTIGATION, EVALUATION AND REHABILITATION OF CONFABULATIONS AFTER BRAIN INJURY**

Participant: ____

Contact person: ____

Contact telephone number: _________________________

Informant person: ____

The purpose of this document is to provide evidence that you or the person representing you, have consented to participate in this intervention program and therefore authorize us to collect and use your information as described in the information sheet. Before signing this document, you should have been informed verbally and written about the investigation.

CONSENT

I declare that I am in conformity with the intervention and the protocol that I have been proposed, and I have successfully received and understood all the information needed to make my decision. Also, I have been informed of my right to withdraw my consent at any time, without obligation to justify my will and without any adverse consequences for me. At that moment, my data would be removed of the study. I also declare that I have been informed of my right to request further additional information in case I need it.

<table>
<tr><td>Signature of the patient:

Date:</td><td>Signature of the representative of the patient:

Date:</td><td>Signature of the informant:

Date:</td></tr>
<tr><td>Representation by:
□ Will of the interested person
□ Minority of age
□ Incapacity of the interested person</td><td colspan="2">SIGNATURE FOR REVOCATION

Name:
Date:</td></tr>
</table>
